# Efficacy and tolerability of aripiprazole versus D_2_ antagonists in the early course of schizophrenia: a systematic review and meta-analysis

**DOI:** 10.1038/s41537-021-00158-z

**Published:** 2021-05-25

**Authors:** David D. Kim, Alasdair M. Barr, Lulu Lian, Jessica W. Y. Yuen, Diane Fredrikson, William G. Honer, Allen E. Thornton, Ric M. Procyshyn

**Affiliations:** 1 Department of Anesthesiology, Pharmacology & Therapeutics, University of British Columbia, Vancouver, Canada; 2 British Columbia Mental Health & Substance Use Services Research Institute, Vancouver, Canada; 3 Department of Psychiatry, University of British Columbia, Vancouver, Canada; 4 Department of Psychology, Simon Fraser University, Burnaby, Canada

**Keywords:** Schizophrenia, Psychosis

## Abstract

Early intervention is essential for favorable long-term outcomes in schizophrenia. However, there is limited guidance in the scientific literature on how best to choose between dopamine D_2_ receptor (D_2_R) partial agonists and D_2_R antagonists in early stages of schizophrenia. The aim of this meta-analysis was to directly compare D_2_R partial agonists with D_2_R antagonists for efficacy and tolerability, using randomized controlled trials (RCTs) that involved participants diagnosed with first-episode psychosis, schizophrenia, or related psychotic disorders with a duration of illness ≤5 years. Fourteen RCTs, involving 2494 patients, were included in the meta-analysis. Aripiprazole was the only identified D_2_R partial agonist, and was not significantly different from pooled D_2_R antagonists for overall symptom reduction or all-cause discontinuation. However, aripiprazole was more favorable than pooled D_2_R antagonists for depressive symptoms, prolactin levels, and triglyceride levels. Specifically, aripiprazole was more favorable than paliperidone for triglyceride levels and more favorable than risperidone and olanzapine, but less favorable than ziprasidone, for weight gain. In addition, aripiprazole was less favorable for akathisia compared with second-generation D_2_R antagonists, in particular olanzapine and quetiapine, and less favorable for discontinuation due to inefficacy than risperidone. Lastly, aripiprazole was more favorable than haloperidol for various efficacy and tolerability outcomes. In conclusion, aripiprazole’s efficacy did not differ substantially from D_2_R antagonists in the early course of schizophrenia, whereas differential tolerability profiles were noted. More double-blind RCTs are required comparing the efficacy and tolerability of aripiprazole as well as other D_2_R partial agonists with D_2_R antagonists in early stages of schizophrenia.

## Introduction

Schizophrenia is a debilitating psychiatric disorder that affects 0.87% of the general population over a lifetime^1^. The onset of schizophrenia is typically between the ages of 14 and 35^2^, and the disease is associated with a reduction in lifespan by approximately 16.3–18.7 years compared with the general population^3^. Recent research shows that antipsychotic treatment is associated with a lower mortality risk compared with no treatment during follow-up periods^4^. This illustrates the importance of utilizing antipsychotics in patients with schizophrenia, but how these medications should be used for optimal outcomes requires further research.

First-episode psychosis (FEP) refers to the first time an individual experiences psychotic symptoms, which gives significant distress, confusion, and fear to many individuals^5^. There is well-established evidence that the duration of untreated psychosis is negatively associated with long-term outcomes, indicating that intervening early and effectively is important in FEP^5^. The literature suggests that an illness duration of less than 5 years is considered an acceptable period of time to define the early stage of schizophrenia^6^. Thus, a person who has FEP, or is in an early stage of schizophrenia, should be provided with evidence-based pharmacotherapy in a timely manner.

Current treatment guidelines recommend second-generation antipsychotics, including risperidone, quetiapine, olanzapine, and aripiprazole, as first-line treatment for schizophrenia^7^. Aripiprazole’s pharmacology is distinct from most antipsychotics in that it is a dopamine D_2_ receptor (D_2_R) partial agonist, rather than a full D_2_ receptor antagonist^8^. Owing to this difference in pharmacology, D_2_R partial agonists (including brexpiprazole and cariprazine) have also been referred to as third-generation antipsychotics^8^. To date, there is limited evidence as to how D_2_R partial agonists differ from D_2_R antagonists in the early course of schizophrenia. The most recent network meta-analysis of the efficacy and tolerability of antipsychotics in FEP included 19 randomized controlled studies (RCTs), of which only one RCT involved a D_2_ partial agonist, aripiprazole^9^.

The small number of RCTs in the literature that compared D_2_ partial agonists with D_2_ antagonists in FEP led us to conduct a systematic review that does not restrict the target population to FEP but also includes all individuals in the early course of their disease, using the evidence-based definition (i.e., a duration of illness less than 5 years)^6^. The objective of our study was to conduct a meta-analysis comparing the efficacy and tolerability of D_2_R partial agonists with D_2_R antagonists in the early course of schizophrenia.

## Results

### Study characteristics

Of the 1072 records identified, 14 studies, involving a total of 2494 patients were included in our meta-analysis (Supplementary Fig. 1)^10–23^. Included studies are summarized in Table 1. Seven of our included studies were open-label (50%), 4 were double-blind (28.6%), and 2 were single-blind RCTs (14.3%). Blinding was unknown for one study (7.1%). In this meta-analysis, aripiprazole was the only D_2_R partial agonist that was identified and all studies utilized an oral form of aripiprazole. Aripiprazole (*n* = 1013) was compared directly with D_2_R antagonists (*n* = 1481), including risperidone (*n* = 421; 7 studies), olanzapine (*n* = 298; 4 studies), quetiapine (*n* = 206; 4 studies), haloperidol (*n* = 209; 3 studies), ziprasidone (*n* = 150; 3 studies), paliperidone (*n* = 175; 2 studies), and perospirone (*n* = 22; 1 study). The pooled mean age of the entire sample (one study missing; *n* = 2434) was 25.85 (SD = 9.19) years and male patients consisted of 60.6% of the population. The mean duration of illness was 1.78 years (*n* = 2302; 10 studies). Regarding the risk of bias assessment, few details were reported about allocation of concealment (14.3%), and less than half of the included studies demonstrated low risk for blinding of participants and personnel (28.6%) and blinding of outcome assessment (42.9%). Other aspects of risk of bias assessment are summarized in Supplementary Fig. 2. Four studies reported results for multiple follow-up durations^11,16,18,23^. For some of the included studies^19,22,23^, outcomes of interest were additionally found in separate publications^24–33^.

**Table 1:** Summary of included studies.

Publication	Study design	Baseline sample size	Trial duration	Mean age (y), sex (% male)	Diagnosis; baseline PANSS total score	First episode or early phase	Mean dose (mg/d)
Girgis et al.^10^ (Mixed)	DB-RCT	ARI (*N* = 237)	52 wk	27.2 (6.3), 62.5%	Schizophrenia (DSM-IV); 94.3 (15.4)	According to doi: ~2.8 y (33.6 mo)	ARI: 28.4 (3.1)
		HAL (*N* = 123)					HAL: 8.6 (1.2)
		Total (*N* = 360)					
Zhang et al.^11^ (China)	OL-RCT	ARI (*N* = 71)	52 wk (of 13, 26, 52 wk)	26.4 (7.5), 61.0%	Schizophrenia (DSM-IV); 88.4 (13.0)	According to authors’ definition (first episode), doi: 2.3 (1.1) mo	ARI: 12.4 (6.5)
		PAL (*N* = 63)					PAL: 6.4 (5.3)
		ZIP (*N* = 69)					ZIP: 36.4 (15.6)
		Total (*N* = 203)					
Maat et al.^12^ (Netherlands)	OL-RCT	ARI (*N* = 20)	8 wk	25.5 (6.0), 81.3%	Schizophrenia (DSM-IV); 71.9 (13.5)	According to authors’ reply (doi: <5 y)	ARI: 17.0 (6.2)
		RIS (*N* = 28)					RIS: 3.6 (1.1)
		Total (*N* = 48)					
Zhang et al.^13^ (China)	SB-RCT	ARI (*N* = 50)	8 wk	41.0 (13.0), 65.3%	Schizophrenia (CCMD-3); 90.5 (14.2)	According to authors’ definition (first-episode, antipsychotic-naïve), doi: 22.5 (18.6) mo	ARI: 16.4 (3.2)
		QUE (*N* = 50)					
		OLA (*N* = 50)					QUE: 598.0 (147.0)
		Total (*N* = 150)					OLA: 18.1 (3.0)
Kuzmanovic et al.^14^ (Serbia)	?-RCT	ARI (*N* = 30)	26 wk	No info	First-episode schizophrenia; 107.2	According to authors’ definition (first episode), doi:?	ARI: 13.2
		HAL (*N* = 30)					HAL: 11.8
		Total (*N* = 60)					
Robinson et al.^15^ (USA & Canada)	DB-RCT	ARI (*N* = 102)	12 wk	22.1 (5.6), 71%	Schizophrenia, schizophreniform, schizoaffective disorder, or psychotic disorder NOS (DSM-IV); ~80	According to authors’ definition (first episode), doi: 125.5 (208.8) wk (28.9 mo)	ARI: 14.8 (6.0)
		RIS (*N* = 96)					RIS: 3.2 (1.5)
		Total (*N* = 198)					
Savitz et al.^16^ (Mixed)	DB-RCT	ARI (*N* = 114)	26 wk (of 8, 26 wk)	15.3 (1.5), 66.0%	Schizophrenia (DSM-IV, K-SADS-PL); 90.8 (12.2)	According to doi: 2.9 (2.1) y (34.8 mo)	ARI: 11.6 (3.0)
		PAL (*N* = 112)					PAL: 6.8 (1.8)
		Total (*N* = 226)					
Takekita et al.^17^ (Japan)	OL-RCT	ARI (*N* = 18)	12 wk	39.8 (16.0), 55.0%	Schizophrenia (DSM-IV); 101.3 (18.2)	According to authors’ definition (antipsychotic-naïve), doi:?	ARI: 14.7 (4.7)
		PER (*N* = 22)					PER: 18.4 (11.6)
		Total (*N* = 40)					
Nussbaum et al.^18^ (Romania)	OL-RCT	ARI (*N* = 22)	104 wk (of 13, 26, 52, 78, 104 wk)	16.0 (2.5), 53.3%	Schizophrenia or bipolar disorder (DSM-IV, K-SADS-PL) - only used schizophrenia; 135.3 (15.9)	According to mean age: <18 y, doi:?	No info
		RIS (*N* = 22)					
		Total (*N* = 44)					
Pagsberg et al.^19^; related publication^31^ (Denmark)	DB-RCT	ARI (*N* = 58)	12 wk	15.8 (1.3), 30.1%	Schizophrenia-spectrum disorder, delusional disorder, affective-spectrum psychotic disorder (ICD-10); 77.9 (12.3)	According to authors’ definition (first episode), doi: 30 mo	ARI: 13.0 (5.5)
		QUE (*N* = 55)					QUE: 426.4 (169.3)
		Total (*N* = 113)					
Wang et al.^20^ (China)	OL-RCT	ARI (*N* = 39)	6-8 wk	27.8 (8.3), 46.3%	Schizophrenia (DSM-IV); ~99	According to authors’ definition (first episode, antipsychotic-naïve), doi: 8.5 (13.0) mo	ARI: 25.7 (6.5)
		RIS (*N* = 43)					RIS: 5.0 (2.5)
		QUE (*N* = 39)					QUE: 634.5 (207.6)
		ZIP (*N* = 19)					ZIP: 102.9 (49.6)
		OLA (*N* = 35)					OLA: 17.6 (3.4)
		Total (*N* = 175)					
Liemburg et al.^21^ (Netherlands)	SB-RCT	ARI (*N* = 12)	9 wk	27.7 (10.0), 87.5%	Schizophrenia or a related non-affective psychotic disorder (DSM-IV); 66.3 (10.6)	According to doi: 3.9 (6.4) y (46.8 mo)	ARI: 7.7 (2.3)
		RIS (*N* = 12)					RIS: 2.3 (1.0)
		Total (*N* = 24)					
Cheng et al.^22^ ; related publications^32,33^ (China)	OL-RCT	ARI (*N* = 162)	8 wk	24.8 (7.4), 50.5%	Schizophrenia (DSM-IV); 86.1 (14.9)	According to authors’ definition (first episode), doi: 8.8 mo	ARI: 17.6 (5.2)
		RIS (*N* = 157)					RIS: 3.7 (1.2)
		OLA (*N* = 158)					OLA: 16.0 (4.9)
		Total (*N* = 477)					
Gómez-Revuelta et al.^23^; related publications^24–30^ (Spain)	OL-RCT	ARI (*N* = 78)	3 yr	29.6 (2.6), 57.5%	Brief psychotic disorder, schizophrenia, schizophreniform, schizoaffective disorder, psychotic disorder NOS (DSM-IV); ~111	According to authors’ definition (first episode), doi: 25.4 (5.9) mo	ARI: 16.3
		RIS (*N* = 63)					RIS: 2.9
		QUE (*N* = 62)					QUE: 195.9
		ZIP (*N* = 62)					ZIP: 121.3
		OLA (*N* = 55)					OLA: 8.7
		HAL (*N* = 56)					HAL: 3.2
		Total (*N* = 376)					

*ARI* aripiprazole, *CCMD* Chinese Classification of Mental Disorders, *DB* double-blind, *DOI* duration of illness, *HAL*: haloperidol, *K-SADS-PL* Kiddie Schedule for Affective Disorders and Schizophrenia for School-Age Children-Present and Lifetime Version, *OL* open-label, *OLA* olanzapine, *PAL* paliperidone, *PANSS* Positive and Negative Syndrome Scale, *PER* perospirone, *QUE* quetiapine, *RCT* randomized controlled trial, *RIS* risperidone, *SB* single-blind, *ZIP* ziprasidone.

### Aripiprazole versus pooled D_2_R antagonists

Results of comparing aripiprazole with D_2_R antagonists (as a group) are shown in Table 2. Aripiprazole was not significantly different from D_2_R antagonists in terms of overall symptom reduction (SMD = −0.05, 95% CI = −0.27 to 0.18; Table 2). In terms of other secondary outcomes, aripiprazole was more favorable than D_2_R antagonists for depressive symptoms (SMD = −0.17, 95% CI = −0.31 to −0.04), prolactin levels (SMD = −0.55, 95% CI = −0.94 to −0.16), and triglyceride levels (SMD = −0.23, 95% CI = −0.45 to −0.02) (Table 2). Removal of haloperidol trials led to aripiprazole being less favorable than D_2_R antagonists for akathisia (RR = 1.42, 95% CI = 1.11 to 1.81) and use of anticholinergics (RR = 1.32, 95% CI = 1.01 to 1.72).

**Table 2:** Comparisons of aripiprazole with D_2_R antagonists in the early course of schizophrenia.

Outcomes	Studies (*n*)	ARI (*n*)	Other (*n*)	Comparator antipsychotics	Effect estimate^a^	*p*	*I*^*2*^
Overall symptoms^b^	14	834	1255	R, Q, O, Z, Pal, Per, H	−0.05 (−0.27 to 0.18)^SMD^	0.69	80%
Short-term trials	12	710	1067	R, Q, O, Z, Pal, Per	0.10 (−0.10 to 0.29)	0.32	69%
Long-term trials	7	427	606	R, Q, O, Z, Pal, H	−0.30 (−0.68 to 0.08)	0.12	86%
Positive symptoms	9	586	901	R, Q, O, Z, Pal, Per, H	−0.03 (−0.23 to 0.17)^SMD^	0.79	63%
Short-term trials	8	492	739	R, Q, O, Z, Pal, Per	0.02 (−0.16 to 0.20)	0.82	50%
Long-term trials	4	304	426	R, Q, O, Z, Pal, H	−0.18 (−0.41 to 0.05)	0.13	46%
Negative symptoms	9	586	901	R, Q, O, Z, Pal, Per, H	−0.00 (−0.15 to 0.15)^SMD^	0.99	37%
Short-term trials	8	492	739	R, Q, O, Z, Pal, Per	0.03 (−0.11 to 0.17)	0.65	22%
Long-term trials	4	304	426	R, Q, O, Z, Pal, H	−0.16 (−0.32 to 0.00)	0.06	0%
Depressive symptoms	6	440	532	R, Q, O, Z, Pal, H	−0.17 (−0.31 to −0.04)^SMD^	0.01	0%
Short-term trials	5	346	370	R, Q, Z, Pal	−0.20 (−0.35 to −0.04)	0.01	10%
Long-term trials	3	280	376	R, Q, O, Z, Pal, H	−0.17 (−0.35 to 0.01)	0.06	8%
All-cause discontinuation	11	1006	1495	R, Q, O, Z, Pal, H	0.92 (0.81 to 1.05)^RR^	0.24	48%
Short-term trials	9	650	1089	R, Q, O, Z, Pal	1.05 (0.76 to 1.45)	0.78	70%
Long-term trials	6	711	1049	R, Q, O, Z, Pal, H	0.88 (0.75 to 1.04)	0.13	62%
Discontinuation due to adverse events	8	891	1227	R, Q, O, Z, Pal, H	0.59 (0.33 to 1.07)^RR^	0.08	65%
Short-term trials	7	589	931	R, Q, O, Z, Pal	0.94 (0.59 to 1.50)	0.81	6%
Long-term trials	5	689	1027	R, Q, O, Z, Pal, H	0.40 (0.19 to 0.83)	0.01^c^	66%
Discontinuation due to inefficacy	7	602	1111	R, Q, O, Z, Pal, H	1.64 (0.77 to 3.50)^RR^	0.20	74%
Short-term trials	6	483	828	R, Q, O, Z, Pal	1.20 (0.53 to 2.72)	0.66	64%
Long-term trials	4	452	904	R, Q, O, Z, Pal, H	1.48 (0.56 to 3.89)	0.42	87%
Use of anticholinergics	8	765	1048	R, Q, O, Z, Pal, Per, H	1.20 (0.87 to 1.65)^RR^	0.26^d^	74%
Short-term trials	6	404	621	R, Q, O, Z, Per	1.45 (0.98 to 2.15)	0.06	58%
Long-term trials	4	453	584	R, Q, O, Z, Pal, H	1.09 (0.65 to 1.85)	0.74	82%
Akathisia	8	647	751	R, Q, O, Z, Pal, Per, H	1.24 (0.88 to 1.74)^RR^	0.21^d^	60%
Short-term trials	6	373	636	R, Q, O, Z, Per, H	1.42 (1.05 to 1.90)	0.02	39%
Long-term trials	3	417	468	R, Q, O, Z, Pal, H	0.80 (0.54 to 1.17)	0.25	28%
Weight gain	8	809	1063	R, Q, O, Z, Pal, Per, H	−0.11 (−0.47 to 0.25)^SMD^	0.54	90%
Short-term trials	7	574	806	R, Q, O, Z, Pal, Per	−0.08 (−0.54 to 0.39)	0.74	94%
Long-term trials	4	488	599	R, Q, O, Z, Pal, H	0.12 (−0.42 to 0.67)	0.65	91%
Total cholesterol	7	447	591	R, Q, O, Z, Pal, Per	−0.20 (−0.47 to 0.06)^SMD^	0.14	70%
Short-term trials	6	382	451	R, Q, O, Z, Pal, Per	−0.28 (−0.49 to −0.06)	0.01	56%
Long-term trials	3	233	330	Q, Z, Pal	0.07 (−0.41 to 0.55)	0.77	76%
Triglycerides	7	448	590	R, Q, O, Z, Pal, Per	−0.23 (−0.45 to −0.02)^SMD^	0.03	55%
Short-term trials	6	375	444	R, Q, O, Z, Pal, Per	−0.27 (−0.42 to −0.11)	<0.001	18%
Long-term trials	3	233	330	Q, Z, Pal	−0.06 (−0.42 to 0.29)	0.73	61%
Glucose	7	449	595	R, Q, O, Z, Pal, Per	−0.06 (−0.31 to 0.20)^SMD^	0.66	69%
Short-term trials	6	383	454	R, Q, O, Z, Pal, Per	−0.22 (−0.35 to −0.08)	0.002	0%
Long-term trials	3	234	333	Q, Z, Pal	0.17 (−0.43 to 0.76)	0.58	87%
Prolactin	5	294	342	R, Q, Z, Pal, Per	−0.55 (−0.94 to −0.16)^SMD^	0.006	81%
Short-term trials	5	295	339	R, Q, Z, Pal, Per	−0.67 (−1.07 to −0.26)	0.001	82%
Long-term trials	2	146	186	Q, Z, Pal	−0.52 (−1.17 to 0.12)	0.11	88%
Sedation	6	594	640	R, Q, O, Z, Pal, Per, H	0.91 (0.69 to 1.21)^RR^	0.53	59%
Short-term trials	4	249	274	R, Q, Z, Per	0.96 (0.71 to 1.30)	0.79	47%
Long-term trials	3	417	468	R, Q, O, Z, Pal, H	0.75 (0.57 to 0.99)	0.04	0%

*D*_2_*R* dopamine *D*_2_ receptor, *EPS* extrapyramidal symptoms, *H* haloperidol, *O* olanzapine, *Pal* paliperidone, *Per* perospirone, *Q* quetiapine, *R* risperidone, *RR* risk ratio, *SMD* standardized mean difference, *Z* ziprasidone.

^a^SMD less than 0 and RR less than 1 indicate aripiprazole is favored compared with other antipsychotics.

^b^Based on either the Positive and Negative Syndrome Scale or other validated scales (e.g., the Brief Psychiatric Rating Scale).

^c^No longer significant after removing haloperidol.

^d^Became significant (aripiprazole being less favorable) after removing haloperidol.

When only blinded RCTs were considered (4 double-blind, 2 single-blind), aripiprazole remained more favorable than D_2_R antagonists for depressive symptoms (*p* = 0.03), prolactin levels (*p* = 0.008), and triglyceride levels (*p* = 0.002). In addition, aripiprazole was more favorable than D_2_R antagonists for total cholesterol (*p* < 0.001) and glucose levels (*p* = 0.001), and less favorable than D_2_R antagonists for discontinuation due to inefficacy (*p* = 0.02). Including only the double-blind RCTs led to aripiprazole being more favorable than D_2_R antagonists for prolactin (*p* = 0.008), triglyceride (*p* = 0.03), total cholesterol (*p* < 0.001), and glucose levels (*p* = 0.001), and less favorable for discontinuation due to inefficacy (*p* = 0.02). Restricting the analysis to open-label RCTs (*N* = 7 trials) led to aripiprazole being less favorable than second-generation D_2_R antagonists for akathisia (*p* = 0.02) and anticholinergic use (*p* < 0.001), whereas no significant difference emerged for blinded RCTs.

When studies were stratified according to trial duration, for short-term trials (<6 months), aripiprazole was significantly more favorable than D_2_R antagonists for depressive symptoms (SMD = −0.20, 95% CI = −0.35 to −0.04), total cholesterol levels (SMD = −0.28, 95% CI = −0.49 to −0.06), triglyceride levels (SMD = −0.27, 95% CI = −0.42 to −0.11), glucose levels (SMD = −0.22, 95% CI = −0.35 to −0.08), and prolactin levels (SMD = −0.67, 95% CI = −1.07 to −0.26), and less favorable for akathisia (RR = 1.42, 95% CI = 1.05 to 1.90). Removal of the short-term trial that used perospirone (which is only used in Japan) did not change the significance of the results. For long-term trials (≥6 months), aripiprazole was significantly more favorable than D_2_R antagonists for discontinuation due to adverse events (RR = 0.40, 95% CI = 0.19 to 0.83) and sedation (RR = 0.75, 95% CI = 0.57 to 0.99). When the long-term trials that used first-generation antipsychotics (i.e., haloperidol) were removed from the analysis, aripiprazole was no longer significantly more favorable than D_2_R antagonists for discontinuation due to adverse events.

### Aripiprazole versus individual D_2_R antagonists

Comparisons of aripiprazole with each of the D_2_R antagonists that led to significant results and were based on at least two comparisons are summarized in Table 3. Aripiprazole was associated with larger reductions in overall and negative symptoms than haloperidol and in depressive symptoms than haloperidol and risperidone. Aripiprazole was more favorable for discontinuation due to any cause and adverse events than haloperidol, but less favorable for discontinuation due to inefficacy than risperidone. Aripiprazole was more favorable for metabolic adverse effects than risperidone (weight gain), paliperidone (triglyceride levels), and olanzapine (weight gain), but less favorable than ziprasidone (weight gain). Aripiprazole was less favorable for extrapyramidal side effects than quetiapine (akathisia) and olanzapine (akathisia and use of anticholinergics).

**Table 3:** Significant results of comparing aripiprazole with individual D_2_R antagonists.

	Outcomes	Studies (*n*)	Participants (*n*)	Effect estimate^a^	*p*	*I*^*2*^
*ARI more favorable than*						
Haloperidol	Overall symptoms	3	305	−0.54 (−0.79 to −0.30)^SMD^	<0.001	0%
	Negative symptoms	2	245	−0.36 (−0.64 to −0.09)^SMD^	0.009	0%
	Depressive symptoms	2	245	−0.41 (−0.68 to −0.13)^SMD^	0.004	0%
	All-cause discontinuation	2	494	0.77 (0.67 to 0.88)^RR^	<0.001	28%
	Discontinuation due to adverse events	2	494	0.37 (0.25 to 0.55)^RR^	<0.001	0%
Risperidone	Depressive symptoms	3	331	−0.32 (−0.53 to −0.10)^SMD^	0.005	0%
	Weight gain	3	614	−0.28 (−0.49 to −0.07)^SMD^	0.009	35%
Paliperidone	Triglycerides	2	338	−0.30 (-0.56 to −0.04)^SMD^	0.02	0%
Olanzapine	Weight gain	2	411	−0.73 (−0.93 to −0.52)^SMD^	<0.001	0%
*ARI less favorable than*						
Risperidone	Discontinuation due to inefficacy	3	562	1.72 (1.06 to 2.80)^RR^	0.03	15%
Quetiapine	Akathisia	3	260	1.42 (1.12 to 1.80)^RR^	0.003	0%
Olanzapine	Use of anticholinergics	3	445	2.28 (1.65 to 3.15)^RR^	<0.001	8%
	Akathisia	2	145	5.86 (1.83 to 18.73)^RR^	0.003	0%
Ziprasidone	Weight gain	2	251	0.66 (0.40 to 0.91)^SMD^	<0.001	0%

*D*_*2*_*R* dopamine D_2_ receptor, *EPS* extrapyramidal symptoms, *SMD* standardized mean difference, *RR* risk ratio.

^a^SMD less than 0 and RR less than 1 indicate aripiprazole is favored compared with other antipsychotics.

Studies comparing aripiprazole with risperidone were sufficient in number to be analyzed in short-term and long-term trials. For overall symptom reduction, aripiprazole did not significantly differ from risperidone in short-term trials (*N* = 7 trials, *n* = 732, SMD = 0.13, 95% CI = −0.13 to 0.39, *p* = 0.34), but demonstrated significantly greater efficacy than risperidone in long-term trials (*N* = 3 trials, *n* = 198; SMD = −0.74, 95% CI = −1.25 to −0.24, *p* = 0.004), and a significant difference was found between the two durations (χ^2^ = 6.44, *p* = 0.01). For positive symptom reduction, aripiprazole was significantly less efficacious than risperidone in short-term trials (*N* = 4 trials, *n* = 444; SMD = 0.25, 95% CI = 0.06 to 0.44, *p* = 0.009), but was not significantly different in long-term trials (*N* = 2 trials, *n* = 158; SMD = −0.40, 95% CI = −0.82 to 0.02, *p* = 0.06), and no significant subgroup difference was found between short-term and long-term trials. For negative symptom reduction, no significant difference between aripiprazole and risperidone was found in neither short-term nor long-term trials, and no subgroup difference between the two durations was noted. For all-cause discontinuation and discontinuation due to adverse events, no significant difference between aripiprazole and risperidone was found in neither short-term nor long-term trials, and no subgroup difference between the two durations was noted. For discontinuation due to inefficacy, aripiprazole was significantly less favorable than risperidone in short-term trials (*N* = 2 trials, *n* = 410; RR = 1.77, 95% CI = 1.13 to 2.76, *p* = 0.01), but was not significantly different in long-term trials (*N* = 2, *n* = 482; RR = 1.66, 95% CI = 0.85 to 3.24, *p* = 0.14), and no subgroup difference between the two durations was noted.

### Heterogeneity

Heterogeneity (*I*^*2*^ > 50%) was present for overall symptom reduction, positive symptom reduction, discontinuation due to adverse events and inefficacy, use of anticholinergics, incidence of akathisia, weight gain, total cholesterol levels, triglyceride levels, fasting glucose levels, prolactin levels, and incidence of sedation (Table 2). The short-term study (i.e., 8 weeks) that compared aripiprazole with risperidone and olanzapine^22^ and the long-term study (i.e., 3 years) that compared aripiprazole with risperidone, quetiapine, olanzapine, ziprasidone, and haloperidol^23^ were each source of heterogeneity for positive symptom reduction, and removal of each study did not change the significance of the result. The long-term study (i.e., 3 years) that compared aripiprazole with quetiapine and ziprasidone^29^ was the source of heterogeneity for triglyceride levels, and removal of the study did not change the significance of the result. The long-term study (i.e., 1 year) that compared aripiprazole with paliperidone and ziprasidone^11^ was the source of heterogeneity for glucose levels, and removal of the study led to aripiprazole being significantly more favorable than D_2_R antagonists (SMD = −0.21, 95% CI = −0.34 to −0.07). The long-term study (i.e., 1 year) that compared aripiprazole with haloperidol^10^ was the source of heterogeneity for incidence of akathisia, and removal of the study led to aripiprazole being significantly less favorable than D_2_R antagonists (RR = 1.39, 95% CI = 1.08 to 1.80). The long-term study (i.e., 3 years) that compared aripiprazole with risperidone, quetiapine, olanzapine, ziprasidone, and haloperidol^23^ and the long-term study (i.e., 1 year) that compared aripiprazole with risperidone and olanzapine^33^ were each source of heterogeneity for discontinuation due to inefficacy, and removal of the former study led to aripiprazole being less favorable than D_2_R antagonists (RR = 2.25, 95% CI = 1.24 to 4.09). The short-term study (i.e., 12 weeks) that compared aripiprazole with quetiapine^19^ and the long-term study (i.e., 3 years) that compared aripiprazole with risperidone, quetiapine, olanzapine, ziprasidone, and haloperidol^23^ were each source of heterogeneity for incidence of sedation, and removal of each study did not change the significance of the result. No single study was the source of heterogeneity for overall symptom reduction, discontinuation due to adverse events, use of anticholinergics, weight gain, total cholesterol levels, and prolactin levels.

### Meta-regression

In our meta-regression analysis, associations of covariates with overall symptom reduction (*n* = 14 studies) and all-cause discontinuation (*n* = 11 studies) were examined (Supplementary Table 1). Aripiprazole’s effect on overall symptom reduction relative to D_2_R antagonists was positively associated with trial duration (estimate = −0.01, *p* = 0.020) and baseline symptom severity (estimate = −0.02, *p* = 0.041). None of the covariates demonstrated significant associations with all-cause discontinuation.

### Publication bias

We assessed publication bias for overall symptom reduction (*n* = 14 studies) and all-cause discontinuation (*n* = 11 studies) (Supplementary Fig. 3). Egger’s test did not indicate substantial asymmetry in the funnel plots for both outcomes (*p* = 0.546 for overall symptom reduction and *p* = 0.917 for all-cause discontinuation).

## Discussion

Our meta-analysis directly compared aripiprazole with D_2_R antagonists for efficacy and tolerability in the early course of schizophrenia. Aripiprazole was the only identified D_2_R partial agonist and was compared with various D_2_R antagonists, including risperidone, paliperidone, quetiapine, olanzapine, ziprasidone, perospirone, and haloperidol. Our results indicate that aripiprazole’s efficacy was comparable to D_2_R antagonists (as a group), however; it was more favorable in terms of depressive symptoms, triglyceride levels, and prolactin levels. When stratified according to trial duration, aripiprazole was additionally more favorable for total cholesterol levels (short-term), glucose levels (short-term), and sedation (long-term), but was less favorable for akathisia (short-term). These results are largely consistent with existing evidence that aripiprazole has antidepressant effects and a lower tendency to cause hyperprolactinemia, metabolic dysregulation, and sedation^34–38^.

Several mechanisms of action that are unique to aripiprazole may explain such results. Aripiprazole’s D_2_ partial agonistic properties have prolactin-sparing effects^34,35^. Its partial agonistic activity at postsynaptic 5-HT_1A_ and 5-HT_2C_ receptors, coupled with desensitization of presynaptic 5-HT_1A_ receptors, would lead to an increased serotonergic tone, which may play a role in improving depressive and anxiety disorders^38,39^. Aripiprazole’s relatively lower affinity for H_1_ receptors compared with other second-generation antipsychotics may explain its lower propensity to induce sedation^35,36^. Aripiprazole’s lack of anticholinergic effects, relatively modest antagonism of H_1_ receptors, and partial agonistic activity at D_2_ and 5-HT_1A_ receptors would explain its favorable metabolic profiles compared with other second-generation antipsychotics, apart from ziprasidone^37^. These results are consistent with existing evidence that both aripiprazole and ziprasidone, have favorable metabolic profiles relative to many other antipsychotics^37^.

It should be noted that aripiprazole (in short-term trials) was more frequently associated with akathisia compared with D_2_ antagonists, especially quetiapine and olanzapine. This is consistent with a recent network meta-analysis involving patients with FEP, where aripiprazole was less favorable than quetiapine and olanzapine for akathisia^9^. Nevertheless, aripiprazole was not associated with higher discontinuation due to adverse events than D_2_R antagonists, including quetiapine and olanzapine, indicating that the severity of akathisia may have been tolerable. Aripiprazole-induced akathisia may be attributed to its pro-serotonergic effects as well as its functional selectivity for D_2_ receptors. In the case of the latter, aripiprazole may be acting as a full antagonist in certain brain regions (e.g., striatum) where there are higher levels of D_2_ receptor expression^35,40^.

Differential results were found compared with a recent network meta-analysis involving patients with multi-episode, chronic schizophrenia^41^. We found that compared with haloperidol, aripiprazole was more favorable in terms of overall, negative, and depressive symptoms and discontinuation due to any cause and adverse events. This is consistent with a recent network meta-analysis involving patients with FEP that found haloperidol to be less efficacious than second-generation antipsychotics^9^. However, aripiprazole’s efficacy was not significantly different from haloperidol’s in patients with multi-episode, chronic schizophrenia^41^. Also, we found that aripiprazole was less favorable than risperidone in terms of discontinuation due to inefficacy, which is consistent with the finding in patients with multi-episode, chronic schizophrenia where risperidone was more efficacious than aripiprazole^41^.

Such differential results between FEP and multi-episode, chronic schizophrenia may be explained by dopamine supersensitivity, which is a key predictor of poor long-term outcomes in schizophrenia^42^. Individuals with multi-episode, chronic schizophrenia are likely to be exposed to chronic D_2_ receptor blockade, increasing the propensity to develop dopamine supersensitivity^42^. Since dopamine supersensitivity is associated with upregulation of D_2_ receptors, D_2_R antagonists with high affinities for the D_2_ receptor may be more effective than antipsychotics with lower affinities to treat the related psychosis, with the exception of clozapine that has a lower affinity but has the potential to reverse dopamine supersensitivity^43^. Although aripiprazole has a very high affinity for the D_2_ receptor (0.34 nM), its action as a partial agonist would predict worsening of psychosis in an environment of dopamine supersensitivity. This is consistent with reports showing that switching patients with chronic schizophrenia to aripiprazole can be less effective or even lead to psychotic worsening^44^.

However, it should be noted that aripiprazole has been shown to prevent and reverse dopamine supersensitivity and thus may be more effective over the long term^45^. In support of this, our sensitivity analysis found that aripiprazole, although less favorable than risperidone for discontinuation due to inefficacy over a short term, was more efficacious than risperidone over a longer term. Although more studies are required to replicate this finding, the data from animal studies and our preliminary analysis may provide a rationale for using aripiprazole prior to D_2_R antagonists in the treatment of FEP.

Our meta-analysis has several limitations to be considered. First, the number of studies included in our meta-analysis was relatively small. Second, many of the included studies were open-label RCTs, which could have increased the risk of performance and detection bias. Third, aripiprazole was the only identified dopamine D_2_R partial agonist. Due to differences in pharmacological activity even among dopamine D_2_R partial agonists (aripiprazole, brexpiprazole, and cariprazine), there needs to be caution when extrapolating the results of our meta-analysis to D_2_R partial agonists other than aripiprazole. Fourth, heterogeneity was present in various outcomes in our meta-analysis. This was expected as D_2_R antagonists have unique receptor profiles and thus may have varying efficacy and tolerability profiles. However, we identified sources of heterogeneity for the majority of the outcomes and addressed this limitation via subgroup, sensitivity, and meta-regression analyses. Lastly, although multiple outcomes were examined in our review, multiple comparisons were not adjusted for. This may have increased the risk of type 1 error. However, given the relatively small number of studies for each outcome, such a statistical adjustment may be more appropriate when more studies become available in the future.

In conclusion, aripiprazole’s efficacy did not differ substantially from D_2_R antagonists in the early course of schizophrenia, whereas it demonstrated greater antidepressant effects than D_2_R antagonists. Differential tolerability profiles were noted between aripiprazole and D_2_R antagonists, where aripiprazole appeared to have general advantages regarding prolactin and triglyceride levels over D_2_R antagonists, but may induce more akathisia. Evidence is still limited to draw strong conclusions. More double-blind RCTs are required to better understand the relative effects of aripiprazole and other D_2_R partial agonists in the early course of schizophrenia.

## Methods

### Search strategy

The systematic review and meta-analysis were conducted according to the guidelines of Preferred Reporting Items for Systematic Reviews and Meta-Analyses (PRISMA) 2009^46^. Pre-registration with the International Prospective Register of Systematic Reviews was not undertaken for our review, but we ensured that no duplicate review is ongoing to date. We searched for RCTs comparing D_2_R partial agonists with D_2_R antagonists in the early course of schizophrenia that were published up to, and including 15 July 2020, using the MEDLINE, EMBASE, and PsycINFO, and ClinicalTrials.gov databases. The following search terms and associated MeSH terms were used: (1) [‘aripiprazole’ OR ‘brexpiprazole’ OR ‘cariprazine’] AND (2) [(‘first episode’ OR ‘first-episode’ OR ‘$naive’ OR ‘early*’ OR ‘recent*’ OR ‘prodrom*’ OR ‘youth*’ OR ‘adolescen*’ OR ‘child*’) AND (‘schizophreni*’ OR ‘schizophrenia spectrum’ OR ‘psychosis’)] AND (3) [(‘random*’) OR ((‘random*’) AND (‘comparative’ OR ‘comparison*’ OR ‘compare*’ OR ‘versus’ OR ‘vs*’ OR ‘open*’)) OR (‘$blind*’)]. We also manually searched the reference lists of all relevant retrieved articles for potential studies eligible for inclusion in our analysis. Two authors (D.D.K. and L.L.) independently screened for relevant articles and any discrepancy was resolved following a discussion between the two authors or with R.M.P. Corresponding authors of the included studies were contacted for any missing data.

### Selection criteria

Studies were included in the quantitative analysis if they enrolled patients who met the diagnostic criteria for schizophrenia or schizophrenia-spectrum disorders and were in their first episode (as identified by authors) or were in the early course of their illness. We defined early stages of illness as follows: (1) FEP, 2) antipsychotic-naïve, and (3) ≤5 years of mean duration of illness^6^. We placed no restriction on language, blinding, publication year, age, sex, ethnicity, settings, or trial duration.

### Data extraction

The following data were extracted: publication year, sample size, age, sex, duration of illness, trial duration, doses of antipsychotics used, baseline symptom severity, change/endpoint scores for overall, positive, negative, and depressive symptoms, incidence of akathisia, sedation, discontinuation due to any cause, adverse events, and inefficacy, use of medications to treat extrapyramidal symptoms (EPS) (i.e., anticholinergics), and changes in body mass index and levels of prolactin, total cholesterol, triglycerides, and fasting glucose.

Mean antipsychotic doses were converted to chlorpromazine equivalents according to methods provided elsewhere^47,48^. Change scores measured using the Positive and Negative Syndrome Scale (PANSS) were the primary efficacy outcome^49^. Other validated scales were considered if the PANSS was not available. If change scores were not available, authors were contacted for the data. Endpoint scores were used if authors did not respond to our request. D.D.K. and L.L. reviewed included studies and supplementary materials, extracted relevant data, and assessed risk of bias using the Cochrane Risk of Bias tool^50^.

### Data analysis

The meta-analysis was performed using Cochrane Review Manager (version 5.4). Using random-effects models, aripiprazole was compared with D_2_R antagonists (as a group) and also with individual D_2_R antagonists. Standardized mean differences (SMDs) and risk ratios (RRs) were calculated for continuous and dichotomous variables, respectively, with 95% confidence intervals (CIs). SMDs less than 0 and RRs less than 1 indicated that aripiprazole was favored compared with D_2_R antagonists. An inverse variance method was used according to the Cochrane guideline when dichotomous and continuous variables needed to be combined. When necessary, online tools were used to calculate an effect size from *F* or *t* statistics or to combine multiple means and standard deviations^51^. Study heterogeneity was quantified using the *I*^*2*^ statistic, where *I*^*2*^ > 50% was considered substantial heterogeneity. If a study provided data for multiple timepoints, our main analysis included the study’s planned duration. A secondary analysis was performed using the short-term (i.e., <6 months) and long-term (i.e., ≥6 months) data separately.

For outcomes that involved at least 10 studies, meta-regression analysis was performed and publication bias was assessed using R^52^. For the meta-regression analysis, we examined the effects of the following covariates: sample size, study year, age, trial duration, aripiprazole dose, baseline symptom severity, proportion of open-label studies, male participants, studies that included FEP patients, and studies that utilized risperidone or olanzapine. The rationale for including the proportion of risperidone or olanzapine as a covariate was based on a recent meta-analysis that has shown that risperidone and olanzapine tend to be more efficacious than other antipsychotics in multi-episode, chronic schizophrenia^41^. Baseline symptom scores measured on scales other than the PANSS were standardized using methods provided elsewhere^53^. Publication bias was assessed using funnel plots, Egger’s regression test, and trim-and-fill procedure^54^.

## Supplementary information

Supplementary Information

## Data Availability

The manuscript reports meta-analytic data based on original studies. Extracted data are available upon request.
