# Supplementary material for: Efficacy and tolerability of aripiprazole versus D2 antagonists in the early course of schizophrenia: a systematic review and meta-analysis
Source: NPJ Schizophr. 2021 May 25;7:29. doi: 10.1038/s41537-021-00158-z (PMC8149726; doi:10.1038/s41537-021-00158-z)
Supplement: Supplementary file 1 — Supplementary Information [file 41537_2021_158_MOESM1_ESM.pdf]

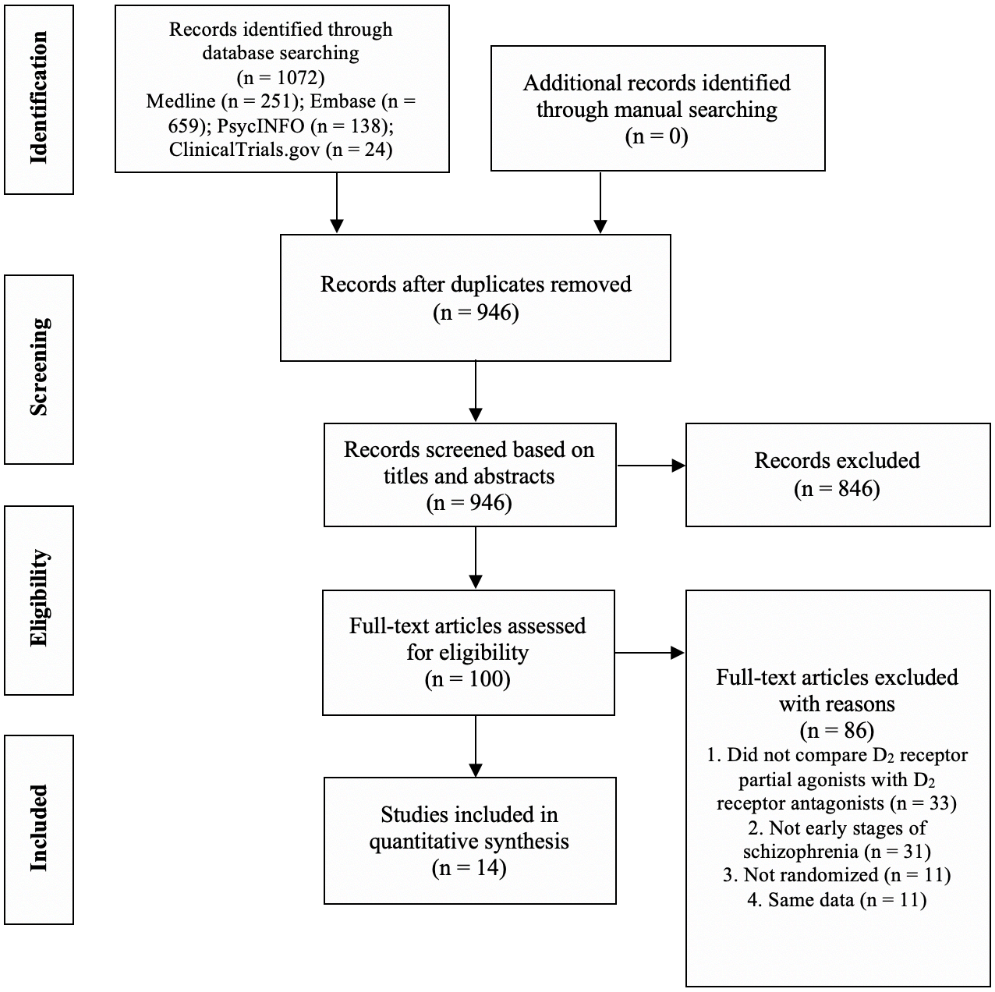

Supplementary Figure 1. PRISMA flow diagram of study selection.

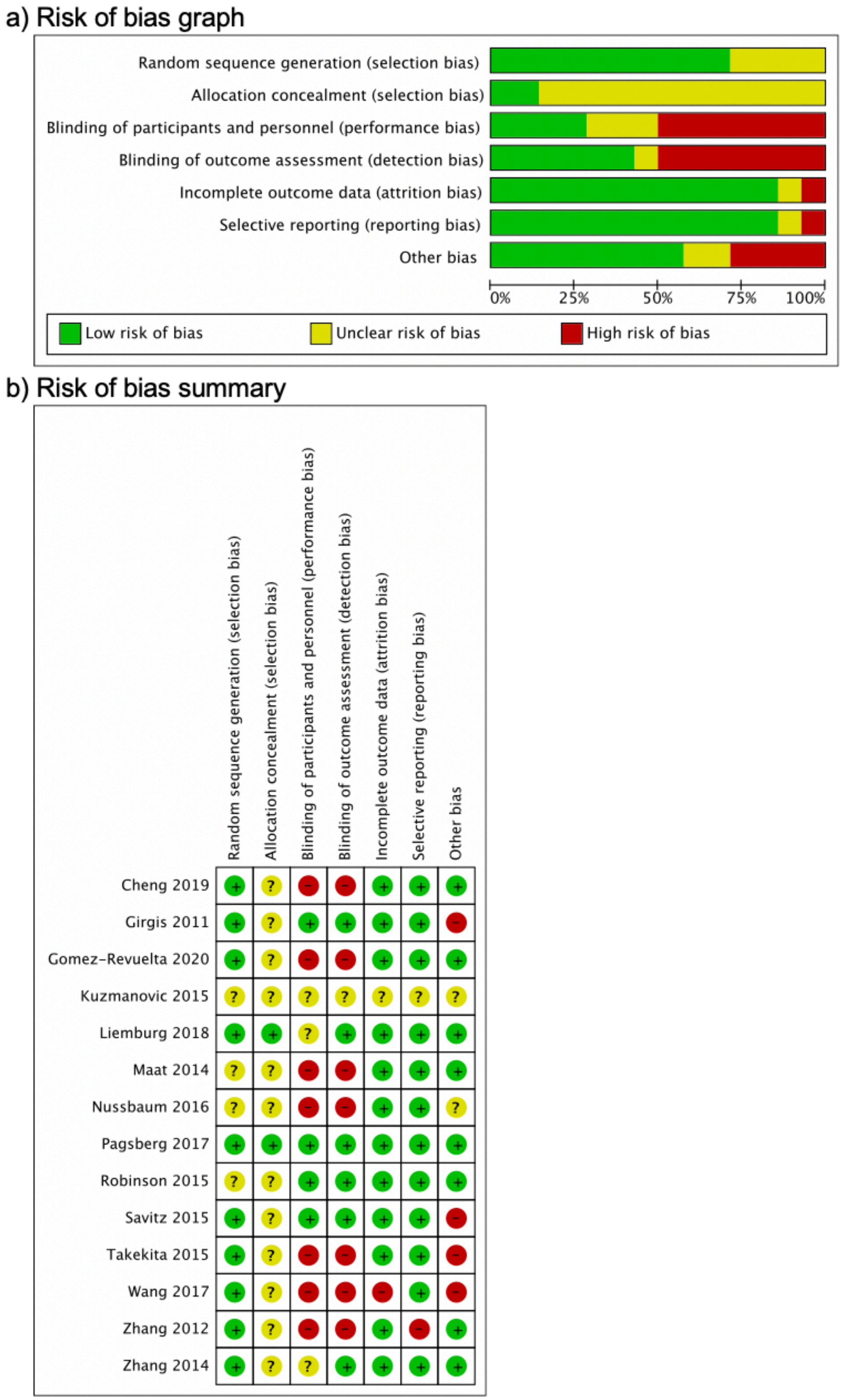

Supplementary Figure 2. Risk of bias assessment.

a) Overall symptoms

Study or Subgroup	Std. Mean Difference	SE	Weight	Std. Mean Difference IV, Random, 95% CI	Year
**1.1.1 All studies**					
Girgis_52wk (H) 2011	−0.45	0.205	7.5%	−0.45 [−0.85, −0.05]	2011
Zhang_52wk (Z+Pal) 2012	0.54	0.1496	8.4%	0.54 [0.25, 0.83]	2012
Maat_8wk (R) 2014	−0.25	0.3109	5.7%	−0.25 [−0.86, 0.36]	2014
Zhang_8wk (Q+O) 2014	0.06	0.1732	8.0%	0.06 [−0.28, 0.40]	2014
Kuzmanovic_26wk (H) 2015	−0.3805	0.2607	6.5%	−0.38 [−0.89, 0.13]	2015
Robinson_12wk (R) 2015	−0.1257	0.1606	8.2%	−0.13 [−0.44, 0.19]	2015
Savitz_26wk (Pal) 2015	−0.07	0.1331	8.7%	−0.07 [−0.33, 0.19]	2015
Takekita_12wk (Per) 2015	0.55	0.3244	5.5%	0.55 [−0.09, 1.19]	2015
Nussbaum_2yr (R) 2016	−1.18	0.3469	5.2%	−1.18 [−1.86, −0.50]	2016
Pagsberg_12wk (Q) 2017	0.02	0.2042	7.5%	0.02 [−0.38, 0.42]	2017
Wang_7wk (R+Q+O+Z) 2017	0.54	0.2404	6.9%	0.54 [0.07, 1.01]	2017
Liemburg_9wk (R) 2018	0.37	0.4124	4.3%	0.37 [−0.44, 1.18]	2018
Cheng_8wk (R+O) 2019	0.22	0.0969	9.2%	0.22 [0.03, 0.41]	2019
Gomez−Revuelta_3yr (R+Q+O+Z+H) 2020	−0.6	0.1417	8.5%	−0.60 [−0.88, −0.32]	2020
**Subtotal (95% CI)**			**100.0%**	**−0.05 [−0.27, 0.18]**	
Heterogeneity: $Tau^2 = 0.13$; $Chi^2 = 65.02$, df = 13 (P < 0.00001); $I^2 = 80\%$					
Test for overall effect: Z = 0.40 (P = 0.69)					
**1.1.2 Short−term studies**					
Zhang_13wk (Z+Pal) 2012	0.54	0.1496	10.3%	0.54 [0.25, 0.83]	2012
Crespo−Facorro_12wk (Q+Z) 2013	−0.51	0.1564	10.1%	−0.51 [−0.82, −0.20]	2013
Maat_8wk (R) 2014	−0.25	0.3109	5.8%	−0.25 [−0.86, 0.36]	2014
Zhang_8wk (Q+O) 2014	0.06	0.1732	9.5%	0.06 [−0.28, 0.40]	2014
Savitz_8wk (Pal) 2015	−0.04	0.1331	10.9%	−0.04 [−0.30, 0.22]	2015
Robinson_12wk (R) 2015	−0.1257	0.1606	10.0%	−0.13 [−0.44, 0.19]	2015
Takekita_12wk (Per) 2015	0.55	0.3244	5.5%	0.55 [−0.09, 1.19]	2015
Nussbaum_13wk (R) 2016	0.08	0.3016	6.0%	0.08 [−0.51, 0.67]	2016
Pagsberg_12wk (Q) 2017	0.02	0.2042	8.6%	0.02 [−0.38, 0.42]	2017
Wang_7wk (R+Q+O+Z) 2017	0.54	0.2404	7.5%	0.54 [0.07, 1.01]	2017
Liemburg_9wk (R) 2018	0.37	0.4124	4.0%	0.37 [−0.44, 1.18]	2018
Cheng_8wk (R+O) 2019	0.22	0.0969	12.0%	0.22 [0.03, 0.41]	2019
**Subtotal (95% CI)**			**100.0%**	**0.10 [−0.10, 0.29]**	
Heterogeneity: $Tau^2 = 0.07$; $Chi^2 = 35.64$, df = 11 (P = 0.0002); $I^2 = 69\%$					
Test for overall effect: Z = 0.98 (P = 0.32)					
**1.1.3 Long−term studies**					
Girgis_52wk (H) 2011	−0.45	0.205	14.5%	−0.45 [−0.85, −0.05]	2011
Zhang_52wk (Z+Pal) 2012	0.54	0.1496	15.7%	0.54 [0.25, 0.83]	2012
Wang_26wk (R+O) 2013	−0.21	0.249	13.5%	−0.21 [−0.70, 0.28]	2013
Kuzmanovic_26wk (H) 2015	−0.3805	0.2607	13.2%	−0.38 [−0.89, 0.13]	2015
Savitz_26wk (Pal) 2015	−0.07	0.1331	16.0%	−0.07 [−0.33, 0.19]	2015
Nussbaum_2yr (R) 2016	−1.18	0.3469	11.1%	−1.18 [−1.86, −0.50]	2016
Gomez−Revuelta_3yr (R+Q+O+Z+H) 2020	−0.6	0.1417	15.9%	−0.60 [−0.88, −0.32]	2020
**Subtotal (95% CI)**			**100.0%**	**−0.30 [−0.68, 0.08]**	
Heterogeneity: $Tau^2 = 0.22$; $Chi^2 = 43.28$, df = 6 (P < 0.00001); $I^2 = 86\%$					
Test for overall effect: Z = 1.53 (P = 0.12)					

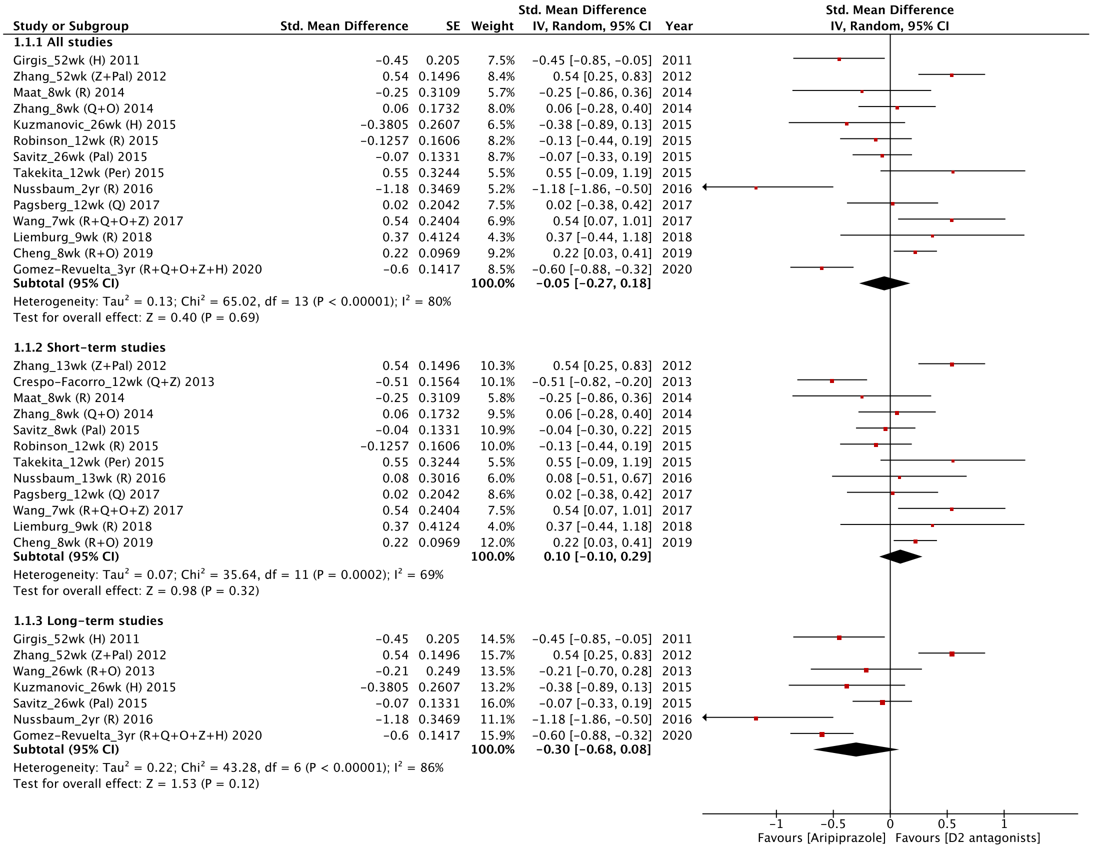

b) All-cause discontinuation

Study or Subgroup	Aripiprazole Events	Aripiprazole Total	Other AP Events	Other AP Total	Weight	Risk Ratio M−H, Random, 95% CI	Year
**1.2.1 All studies**							
Hou (R+O)_52wk 2020	126	169	247	329	22.9%	0.99 [0.89, 1.11]	
Girgis_52wk (H) 2011	123	237	89	123	19.0%	0.72 [0.61, 0.85]	2011
Zhang_52wk (Z+Pal) 2012	19	90	32	164	5.4%	1.08 [0.65, 1.79]	2012
Maat_8wk (R) 2014	18	38	14	42	4.8%	1.42 [0.83, 2.45]	2014
Zhang_8wk (Q+O) 2014	4	54	10	110	1.3%	0.81 [0.27, 2.48]	2014
Savitz_26wk (Pal) 2015	26	115	28	113	6.2%	0.91 [0.57, 1.45]	2015
Robinson_12wk (R) 2015	34	106	40	103	8.7%	0.83 [0.57, 1.19]	2015
Nussbaum_2yr (R) 2016	0	22	4	22	0.2%	0.11 [0.01, 1.95]	2016
Wang_7wk (R+Q+O+Z) 2017	16	39	58	136	7.1%	0.96 [0.63, 1.47]	2017
Pagsberg_12wk (Q) 2017	20	58	12	55	3.9%	1.58 [0.86, 2.92]	2017
Gomez−Revuelta_3yr (R+Q+O+Z+H) 2020	57	78	241	298	20.3%	0.90 [0.78, 1.05]	2020
**Subtotal (95% CI)**		**1006**		**1495**	**100.0%**	**0.92 [0.81, 1.05]**	
Total events	443		775				
Heterogeneity: $Tau^2 = 0.02$; $Chi^2 = 19.16$, df = 10 (P = 0.04); $I^2 = 48\%$							
Test for overall effect: Z = 1.17 (P = 0.24)							
**1.2.2 Short−term studies**							
Zhang_13wk (Z+Pal) 2012	11	90	13	164	9.3%	1.54 [0.72, 3.30]	2012
Crespo−Facorro_12wk (Q+Z) 2013	18	78	61	124	14.2%	0.47 [0.30, 0.73]	2013
Zhang_8wk (Q+O) 2014	4	54	10	110	5.9%	0.81 [0.27, 2.48]	2014
Maat_8wk (R) 2014	18	38	14	42	12.5%	1.42 [0.83, 2.45]	2014
Robinson_12wk (R) 2015	34	106	40	103	15.5%	0.83 [0.57, 1.19]	2015
Nussbaum_13wk (R) 2016	0	22	0	22		Not estimable	2016
Wang_7wk (R+Q+O+Z) 2017	16	39	58	136	14.6%	0.96 [0.63, 1.47]	2017
Pagsberg_12wk (Q) 2017	20	58	12	55	11.4%	1.58 [0.86, 2.92]	2017
Cheng_8wk (R+O) 2019	52	165	71	333	16.6%	1.48 [1.09, 2.01]	2019
**Subtotal (95% CI)**		**650**		**1089**	**100.0%**	**1.05 [0.76, 1.45]**	
Total events	173		279				
Heterogeneity: $Tau^2 = 0.14$; $Chi^2 = 23.45$, df = 7 (P = 0.001); $I^2 = 70\%$							
Test for overall effect: Z = 0.28 (P = 0.78)							
**1.2.3 Long−term studies**							
Hou (R+O)_52wk 2020	126	169	247	329	30.4%	0.99 [0.89, 1.11]	
Girgis_52wk (H) 2011	123	237	89	123	25.7%	0.72 [0.61, 0.85]	2011
Zhang_52wk (Z+Pal) 2012	19	90	32	164	7.7%	1.08 [0.65, 1.79]	2012
Savitz_26wk (Pal) 2015	26	115	28	113	8.7%	0.91 [0.57, 1.45]	2015
Nussbaum_2yr (R) 2016	0	22	4	22	0.3%	0.11 [0.01, 1.95]	2016
Gomez−Revuelta_3yr (R+Q+O+Z+H) 2020	57	78	241	298	27.3%	0.90 [0.78, 1.05]	2020
**Subtotal (95% CI)**		**711**		**1049**	**100.0%**	**0.88 [0.75, 1.04]**	
Total events	351		641				
Heterogeneity: $Tau^2 = 0.02$; $Chi^2 = 13.26$, df = 5 (P = 0.02); $I^2 = 62\%$							
Test for overall effect: Z = 1.53 (P = 0.13)							

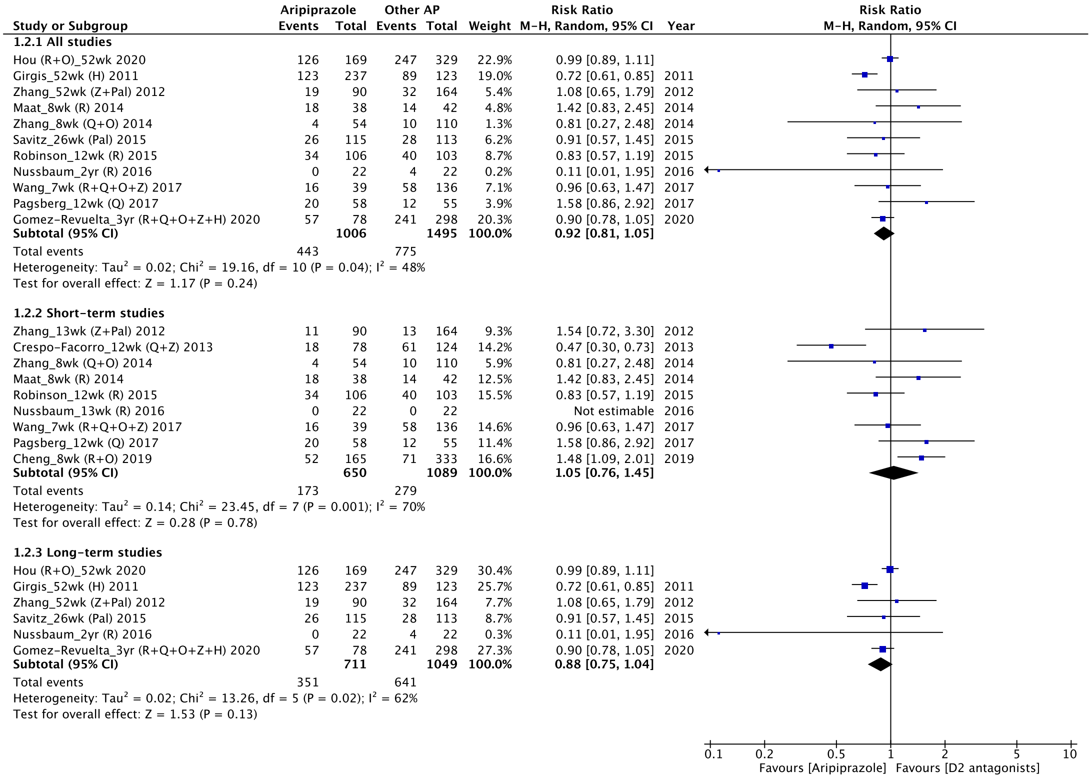

c) Depressive symptoms

Study or Subgroup	Weight	Std. Mean Difference IV, Random, 95% CI	Year
**2.1.1 All studies**			
Girgis_52wk (H) 2011	11.4%	−0.42 [−0.82, −0.02]	2011
Savitz_26wk (Pal) 2015	26.9%	−0.06 [−0.32, 0.20]	2015
Robinson_12wk (R) 2015	23.4%	−0.27 [−0.55, 0.01]	2015
Pagsberg_12wk (Q) 2017	11.4%	0.04 [−0.36, 0.44]	2017
Liemburg_9wk (R) 2018	2.6%	−0.30 [−1.14, 0.54]	2018
Gomez-Revuelta_3yr (R+Q+O+Z+H) 2020	24.4%	−0.17 [−0.44, 0.10]	2020
**Subtotal (95% CI)**	**100.0%**	**−0.17 [−0.31, −0.04]**	
Heterogeneity: Tau² = 0.00; Chi² = 3.79, df = 5 (P = 0.58); I² = 0%			
Test for overall effect: Z = 2.48 (P = 0.01)			
**2.1.2 Short-term studies**			
Crespo-Facorro_12wk (Q+Z) 2013	23.7%	−0.41 [−0.71, −0.11]	2013
Robinson_12wk (R) 2015	27.5%	−0.27 [−0.55, 0.01]	2015
Savitz_8wk (Pal) 2015	31.0%	−0.07 [−0.33, 0.19]	2015
Pagsberg_12wk (Q) 2017	14.5%	0.04 [−0.36, 0.44]	2017
Liemburg_9wk (R) 2018	3.5%	−0.30 [−1.14, 0.54]	2018
**Subtotal (95% CI)**	**100.0%**	**−0.20 [−0.35, −0.04]**	
Heterogeneity: Tau² = 0.00; Chi² = 4.43, df = 4 (P = 0.35); I² = 10%			
Test for overall effect: Z = 2.44 (P = 0.01)			
**2.1.3 Long-term studies**			
Girgis_52wk (H) 2011	19.0%	−0.42 [−0.82, −0.02]	2011
Savitz_26wk (Pal) 2015	42.3%	−0.06 [−0.32, 0.20]	2015
Gomez-Revuelta_3yr (R+Q+O+Z+H) 2020	38.7%	−0.17 [−0.44, 0.10]	2020
**Subtotal (95% CI)**	**100.0%**	**−0.17 [−0.35, 0.01]**	
Heterogeneity: Tau² = 0.00; Chi² = 2.17, df = 2 (P = 0.34); I² = 8%			
Test for overall effect: Z = 1.87 (P = 0.06)			

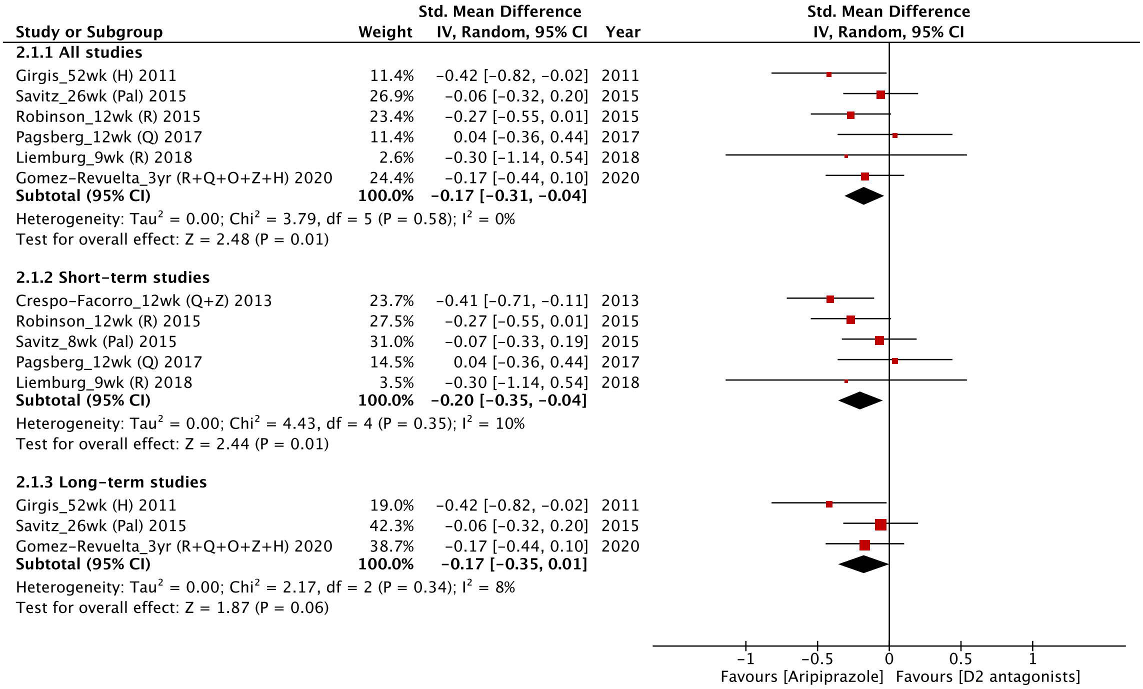

d) Prolactin levels

Study or Subgroup	Weight	Std. Mean Difference IV, Random, 95% CI	Year
**1.2.1 All studies**			
Crespo-Facorro_52wk (Q+Z) 2013	21.6%	−0.19 [−0.53, 0.15]	2013
Robinson_12wk (R) 2015	22.4%	−1.01 [−1.31, −0.72]	2015
Savitz_26wk (Pal) 2015	22.4%	−0.85 [−1.14, −0.55]	2015
Takekita_12wk (Per) 2015	15.3%	−0.59 [−1.22, 0.05]	2015
Pagsberg_12wk (Q) 2017	18.4%	−0.02 [−0.51, 0.47]	2017
**Subtotal (95% CI)**	**100.0%**	**−0.55 [−0.94, −0.16]**	
Heterogeneity: Tau² = 0.15; Chi² = 21.04, df = 4 (P = 0.0003); I² = 81%			
Test for overall effect: Z = 2.76 (P = 0.006)			
**1.2.2 Short-term studies**			
Crespo-Facorro_12wk (Q+Z) 2013	21.5%	−0.42 [−0.76, −0.08]	2013
Savitz_8wk (Pal) 2015	22.1%	−1.16 [−1.47, −0.85]	2015
Takekita_12wk (Per) 2015	15.6%	−0.59 [−1.22, 0.05]	2015
Robinson_12wk (R) 2015	22.3%	−1.01 [−1.31, −0.72]	2015
Pagsberg_12wk (Q) 2017	18.5%	−0.02 [−0.51, 0.47]	2017
**Subtotal (95% CI)**	**100.0%**	**−0.67 [−1.07, −0.26]**	
Heterogeneity: Tau² = 0.17; Chi² = 22.16, df = 4 (P = 0.0002); I² = 82%			
Test for overall effect: Z = 3.23 (P = 0.001)			
**1.2.3 Long-term studies**			
Crespo-Facorro_52wk (Q+Z) 2013	49.2%	−0.19 [−0.53, 0.15]	2013
Savitz_26wk (Pal) 2015	50.8%	−0.85 [−1.14, −0.55]	2015
**Subtotal (95% CI)**	**100.0%**	**−0.52 [−1.17, 0.12]**	
Heterogeneity: Tau² = 0.19; Chi² = 8.25, df = 1 (P = 0.004); I² = 88%			
Test for overall effect: Z = 1.59 (P = 0.11)			

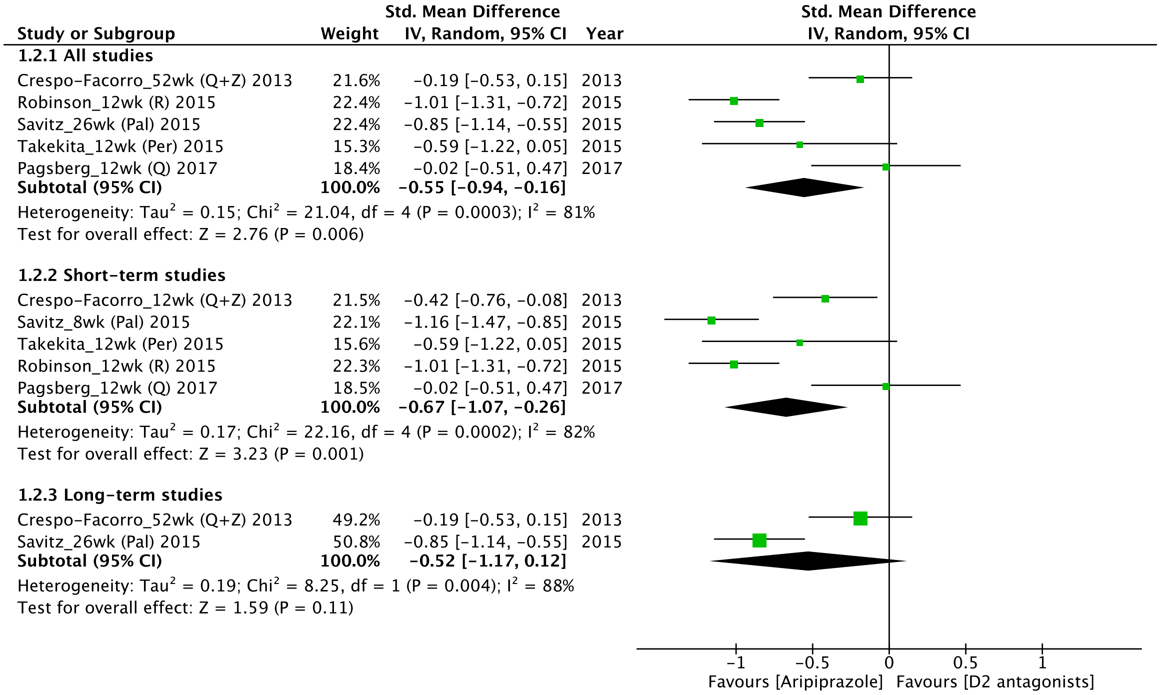

e) Triglyceride levels

Study or Subgroup	Weight	Std. Mean Difference IV, Random, 95% CI	Year
**2.3.1 All studies**			
Zhang_52wk (Z+Pal) 2012	7.6%	−0.18 [−0.84, 0.49]	2012
Zhang_8wk (Q+O) 2014	16.2%	−0.45 [−0.79, −0.11]	2014
Takekita_12wk (Per) 2015	8.2%	−0.35 [−0.98, 0.28]	2015
Savitz_26wk (Pal) 2015	19.0%	−0.27 [−0.55, 0.01]	2015
Robinson_12wk (R) 2015	18.9%	−0.11 [−0.39, 0.17]	2015
Pagsberg_12wk (Q) 2017	13.1%	−0.64 [−1.07, −0.21]	2017
Vazquez-Bourgon_3yr (Q+Z) 2020	17.0%	0.22 [−0.10, 0.54]	2020
**Subtotal (95% CI)**	**100.0%**	**−0.23 [−0.45, −0.02]**	
Heterogeneity: $Tau^2 = 0.04$; $Chi^2 = 13.31$, df = 6 (P = 0.04); $I^2 = 55\%$			
Test for overall effect: Z = 2.13 (P = 0.03)			
**2.3.2 Short-term studies**			
Crespo-Facorro_12wk (Q+Z) 2013	18.0%	−0.18 [−0.51, 0.15]	2013
Zhang_8wk (Q+O) 2014	17.1%	−0.45 [−0.79, −0.11]	2014
Takekita_12wk (Per) 2015	5.8%	−0.35 [−0.98, 0.28]	2015
Savitz_8wk (Pal) 2015	23.8%	−0.16 [−0.44, 0.12]	2015
Robinson_12wk (R) 2015	23.7%	−0.11 [−0.39, 0.17]	2015
Pagsberg_12wk (Q) 2017	11.6%	−0.64 [−1.07, −0.21]	2017
**Subtotal (95% CI)**	**100.0%**	**−0.27 [−0.42, −0.11]**	
Heterogeneity: $Tau^2 = 0.01$; $Chi^2 = 6.06$, df = 5 (P = 0.30); $I^2 = 18\%$			
Test for overall effect: Z = 3.35 (P = 0.0008)			
**2.3.3 Long-term studies**			
Zhang_52wk (Z+Pal) 2012	19.2%	−0.18 [−0.84, 0.49]	2012
Savitz_26wk (Pal) 2015	42.3%	−0.27 [−0.55, 0.01]	2015
Vazquez-Bourgon_3yr (Q+Z) 2020	38.5%	0.22 [−0.10, 0.54]	2020
**Subtotal (95% CI)**	**100.0%**	**−0.06 [−0.42, 0.29]**	
Heterogeneity: $Tau^2 = 0.06$; $Chi^2 = 5.18$, df = 2 (P = 0.07); $I^2 = 61\%$			
Test for overall effect: Z = 0.35 (P = 0.73)			

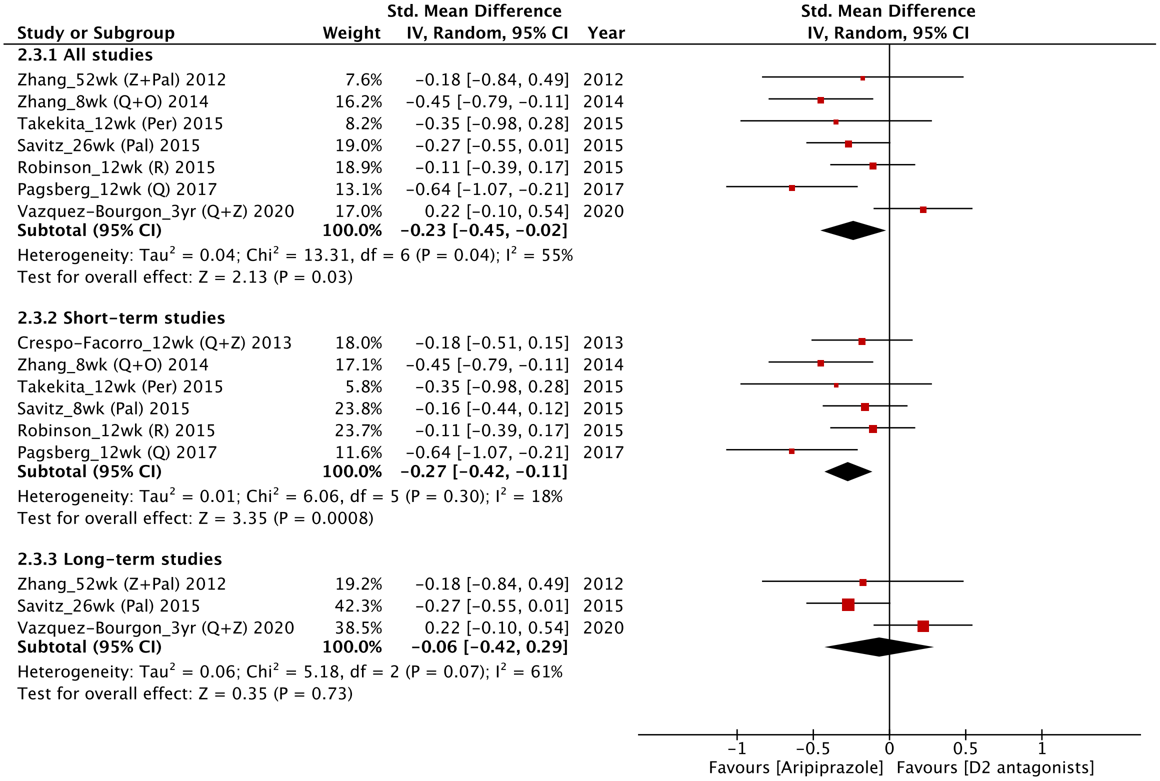

f) Total cholesterol levels

Study or Subgroup	Weight	Std. Mean Difference IV, Random, 95% CI	Year
**2.4.1 All studies**			
Zhang_52wk (Z+Pal) 2012	7.3%	0.65 [−0.16, 1.46]	2012
Zhang_8wk (Q+O) 2014	16.3%	−0.24 [−0.58, 0.10]	2014
Savitz_26wk (Pal) 2015	17.9%	−0.32 [−0.60, −0.04]	2015
Takekita_12wk (Per) 2015	10.0%	−0.01 [−0.63, 0.61]	2015
Robinson_12wk (R) 2015	17.8%	−0.39 [−0.67, −0.11]	2015
Pagsberg_12wk (Q) 2017	13.9%	−0.82 [−1.26, −0.38]	2017
Vazquez-Bourgon_3yr (Q+Z) 2020	16.7%	0.18 [−0.14, 0.50]	2020
**Subtotal (95% CI)**	**100.0%**	**−0.20 [−0.47, 0.06]**	
Heterogeneity: $Tau^2 = 0.08$; $Chi^2 = 19.80$, df = 6 (P = 0.003); $I^2 = 70\%$			
Test for overall effect: Z = 1.49 (P = 0.14)			
**2.4.2 Short-term studies**			
Crespo-Facorro_12wk (Q+Z) 2013	18.8%	0.04 [−0.27, 0.35]	2013
Zhang_8wk (Q+O) 2014	17.6%	−0.24 [−0.58, 0.10]	2014
Savitz_8wk (Pal) 2015	20.6%	−0.23 [−0.51, 0.05]	2015
Takekita_12wk (Per) 2015	8.7%	−0.01 [−0.63, 0.61]	2015
Robinson_12wk (R) 2015	20.5%	−0.39 [−0.67, −0.11]	2015
Pagsberg_12wk (Q) 2017	13.7%	−0.82 [−1.26, −0.38]	2017
**Subtotal (95% CI)**	**100.0%**	**−0.28 [−0.49, −0.06]**	
Heterogeneity: $Tau^2 = 0.04$; $Chi^2 = 11.24$, df = 5 (P = 0.05); $I^2 = 56\%$			
Test for overall effect: Z = 2.49 (P = 0.01)			
**2.4.3 Long-term studies**			
Zhang_52wk (Z+Pal) 2012	20.3%	0.65 [−0.16, 1.46]	2012
Savitz_26wk (Pal) 2015	40.8%	−0.32 [−0.60, −0.04]	2015
Vazquez-Bourgon_3yr (Q+Z) 2020	38.9%	0.18 [−0.14, 0.50]	2020
**Subtotal (95% CI)**	**100.0%**	**0.07 [−0.41, 0.55]**	
Heterogeneity: $Tau^2 = 0.13$; $Chi^2 = 8.50$, df = 2 (P = 0.01); $I^2 = 76\%$			
Test for overall effect: Z = 0.29 (P = 0.77)			

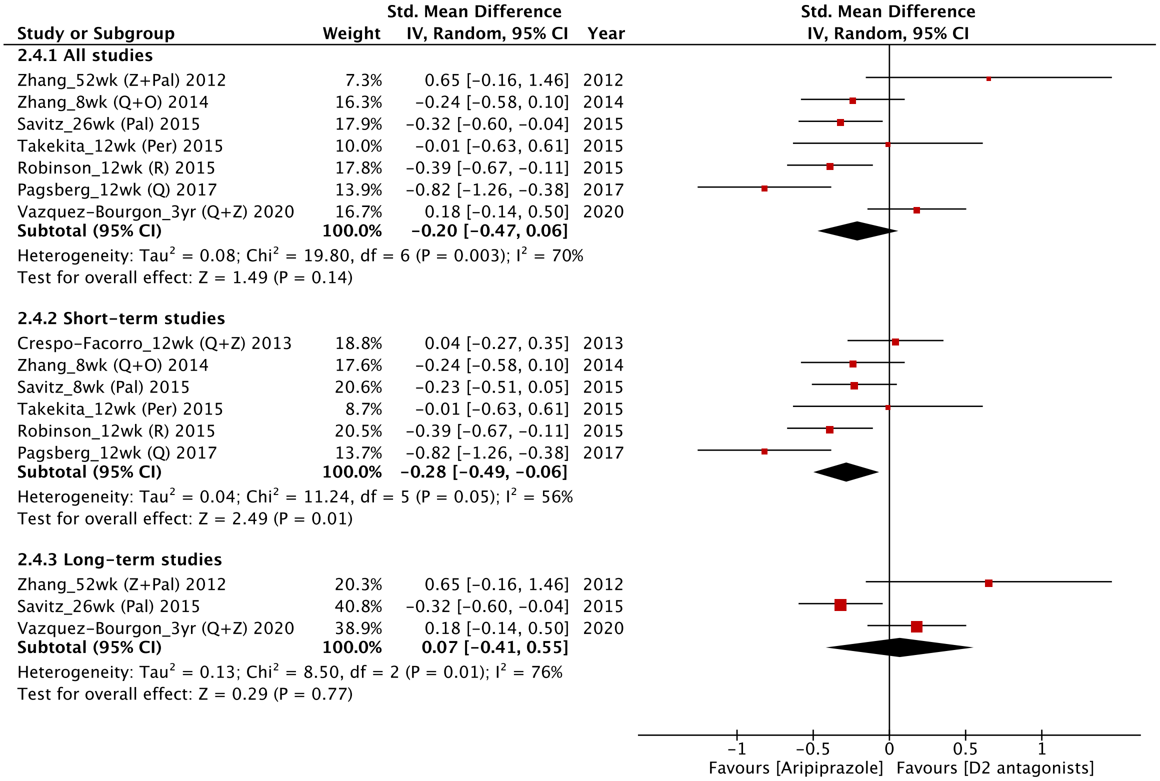

g) Glucose levels

Study or Subgroup	Weight	Std. Mean Difference IV, Random, 95% CI	Year
**2.5.1 All studies**			
Zhang_52wk (Z+Pal) 2012	10.0%	1.01 [0.42, 1.60]	2012
Zhang_8wk (Q+O) 2014	15.8%	−0.14 [−0.48, 0.20]	2014
Savitz_26wk (Pal) 2015	17.5%	−0.29 [−0.56, −0.02]	2015
Takekita_12wk (Per) 2015	9.4%	0.04 [−0.58, 0.66]	2015
Robinson_12wk (R) 2015	17.3%	−0.38 [−0.66, −0.10]	2015
Pagsberg_12wk (Q) 2017	13.8%	−0.12 [−0.54, 0.30]	2017
Vazquez-Bourgon_3yr (Q+Z) 2020	16.2%	−0.04 [−0.36, 0.28]	2020
**Subtotal (95% CI)**	**100.0%**	**−0.06 [−0.31, 0.20]**	
Heterogeneity: Tau² = 0.08; Chi² = 19.27, df = 6 (P = 0.004); I² = 69%			
Test for overall effect: Z = 0.44 (P = 0.66)			
**2.5.2 Short-term studies**			
Crespo-Facorro_12wk (Q+Z) 2013	18.8%	−0.21 [−0.53, 0.11]	2013
Zhang_8wk (Q+O) 2014	16.4%	−0.14 [−0.48, 0.20]	2014
Robinson_12wk (R) 2015	24.0%	−0.38 [−0.66, −0.10]	2015
Savitz_8wk (Pal) 2015	24.9%	−0.21 [−0.49, 0.07]	2015
Takekita_12wk (Per) 2015	4.9%	0.04 [−0.58, 0.66]	2015
Pagsberg_12wk (Q) 2017	11.0%	−0.12 [−0.54, 0.30]	2017
**Subtotal (95% CI)**	**100.0%**	**−0.22 [−0.35, −0.08]**	
Heterogeneity: Tau² = 0.00; Chi² = 2.35, df = 5 (P = 0.80); I² = 0%			
Test for overall effect: Z = 3.09 (P = 0.002)			
**2.5.3 Long-term studies**			
Zhang_52wk (Z+Pal) 2012	28.4%	1.01 [0.42, 1.60]	2012
Savitz_26wk (Pal) 2015	36.4%	−0.29 [−0.56, −0.02]	2015
Vazquez-Bourgon_3yr (Q+Z) 2020	35.3%	−0.04 [−0.36, 0.28]	2020
**Subtotal (95% CI)**	**100.0%**	**0.17 [−0.43, 0.76]**	
Heterogeneity: Tau² = 0.23; Chi² = 15.39, df = 2 (P = 0.0005); I² = 87%			
Test for overall effect: Z = 0.55 (P = 0.58)			

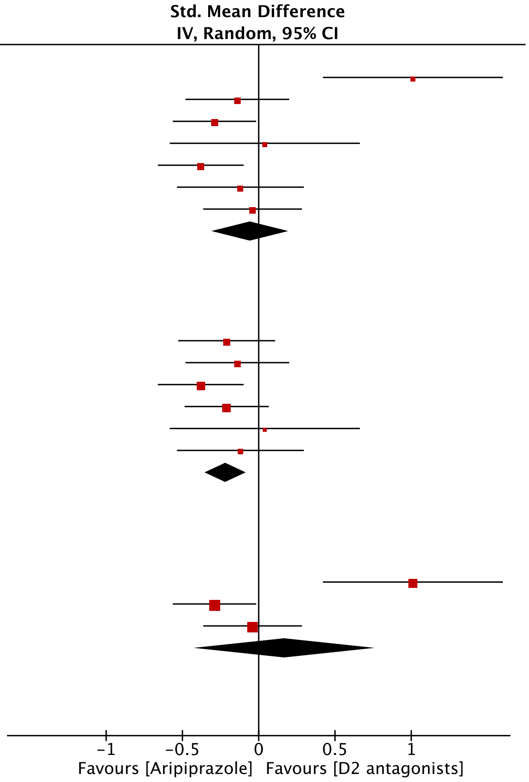

h) Sedation

Study or Subgroup	Weight	Risk Ratio M-H, Random, 95% CI	Year
**1.6.1 All studies**			
Girgis_52wk (H) 2011	9.0%	0.81 [0.36, 1.81]	2011
Takekita_12wk (Per) 2015	3.8%	0.92 [0.23, 3.58]	2015
Robinson_12wk (R) 2015	19.7%	0.94 [0.62, 1.44]	2015
Savitz_8wk (Pal) 2015	9.9%	0.99 [0.47, 2.11]	2015
Pagsberg_12wk (Q) 2017	33.3%	1.09 [0.97, 1.23]	2017
Gomez-Revuelta_3yr (R+Q+O+Z+H) 2020	24.3%	0.71 [0.51, 0.98]	2020
**Subtotal (95% CI)**	**100.0%**	**0.91 [0.69, 1.21]**	
Total events			
Heterogeneity: Tau² = 0.06; Chi² = 12.30, df = 5 (P = 0.03); I² = 59%			
Test for overall effect: Z = 0.63 (P = 0.53)			
**1.6.2 Short-term studies**			
Crespo-Facorro_12wk (Q+Z) 2013	16.9%	0.68 [0.37, 1.26]	2013
Robinson_12wk (R) 2015	26.9%	0.94 [0.62, 1.44]	2015
Takekita_12wk (Per) 2015	4.6%	0.92 [0.23, 3.58]	2015
Pagsberg_12wk (Q) 2017	51.7%	1.09 [0.97, 1.23]	2017
**Subtotal (95% CI)**	**100.0%**	**0.96 [0.71, 1.30]**	
Total events			
Heterogeneity: Tau² = 0.04; Chi² = 5.63, df = 3 (P = 0.13); I² = 47%			
Test for overall effect: Z = 0.27 (P = 0.79)			
**1.6.3 Long-term studies**			
Girgis_52wk (H) 2011	11.8%	0.81 [0.36, 1.81]	2011
Savitz_8wk (Pal) 2015	13.4%	0.99 [0.47, 2.11]	2015
Gomez-Revuelta_3yr (R+Q+O+Z+H) 2020	74.8%	0.71 [0.51, 0.98]	2020
**Subtotal (95% CI)**	**100.0%**	**0.75 [0.57, 0.99]**	
Total events			
Heterogeneity: Tau² = 0.00; Chi² = 0.69, df = 2 (P = 0.71); I² = 0%			
Test for overall effect: Z = 2.01 (P = 0.04)			

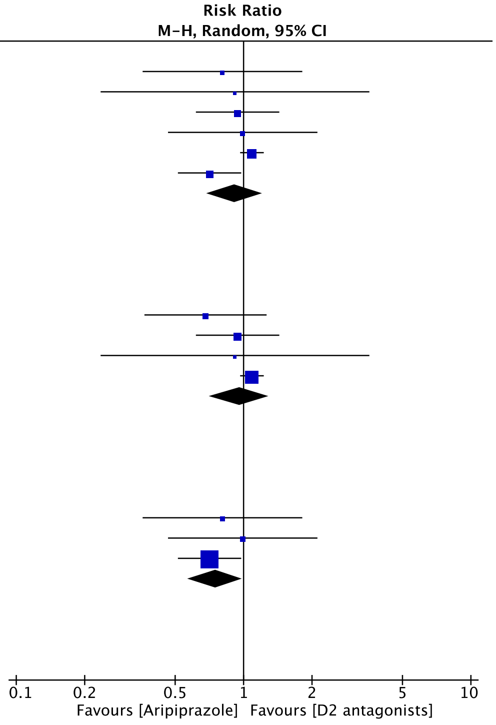

i) Anticholinergic use

Study or Subgroup	Weight	Risk Ratio M-H, Random, 95% CI	Year
**1.7.1 All studies**			
Savitz_26wk (Pal) 2015	16.5%	0.99 [0.64, 1.55]	2015
Robinson_12wk (R) 2015	18.1%	0.81 [0.54, 1.20]	2015
Takekita_12wk (Per) 2015	3.4%	0.92 [0.23, 3.58]	2015
Wang_7wk (R+Q+O+Z) 2017	8.3%	2.12 [0.96, 4.68]	2017
Pagsberg_12wk (Q) 2017	5.3%	3.32 [1.16, 9.47]	2017
Cheng (R+O) 2019	22.5%	1.54 [1.16, 2.06]	2019
Gomez-Revuelta_3yr (R+Q+O+Z) 2020	25.9%	1.46 [1.19, 1.78]	2020
**Subtotal (95% CI)**	**100.0%**	**1.32 [1.01, 1.72]**	
Total events			
Heterogeneity: Tau^2 = 0.06; Chi^2 = 14.05, df = 6 (P = 0.03); I^2 = 57%			
Test for overall effect: Z = 2.05 (P = 0.04)			
**1.7.2 Short-term studies**			
Crespo-Facorro_12wk (Q+Z) 2013	16.4%	1.69 [0.85, 3.38]	2013
Robinson_12wk (R) 2015	24.7%	0.81 [0.54, 1.20]	2015
Takekita_12wk (Per) 2015	6.6%	0.92 [0.23, 3.58]	2015
Pagsberg_12wk (Q) 2017	9.9%	3.32 [1.16, 9.47]	2017
Wang_7wk (R+Q+O+Z) 2017	14.2%	2.12 [0.96, 4.68]	2017
Cheng (R+O) 2019	28.2%	1.54 [1.16, 2.06]	2019
**Subtotal (95% CI)**	**100.0%**	**1.45 [0.98, 2.15]**	
Total events			
Heterogeneity: Tau^2 = 0.12; Chi^2 = 12.02, df = 5 (P = 0.03); I^2 = 58%			
Test for overall effect: Z = 1.87 (P = 0.06)			
**1.7.3 Long-term studies**			
Wang_26wk (R+O) 2013	11.0%	1.85 [0.82, 4.20]	2013
Savitz_26wk (Pal) 2015	28.5%	0.99 [0.64, 1.55]	2015
Gomez-Revuelta_3yr (R+Q+O+Z) 2020	60.5%	1.46 [1.19, 1.78]	2020
**Subtotal (95% CI)**	**100.0%**	**1.34 [1.00, 1.79]**	
Total events			
Heterogeneity: Tau^2 = 0.03; Chi^2 = 3.06, df = 2 (P = 0.22); I^2 = 35%			
Test for overall effect: Z = 1.97 (P = 0.05)			

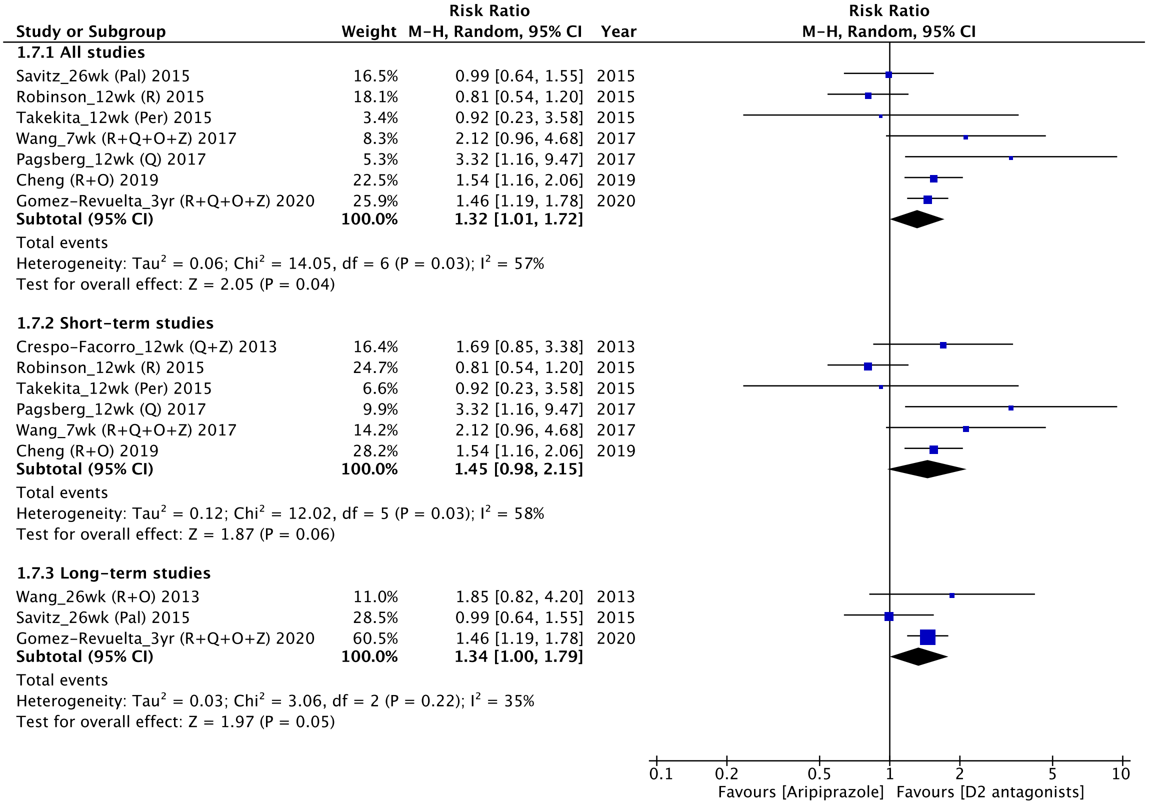

j) Akathisia

Study or Subgroup	Weight	Risk Ratio M-H, Random, 95% CI	Year
**1.8.1 All studies**			
Maat (R) 2014	0.7%	9.92 [0.55, 178.45]	2014
Savitz_26wk (Pal) 2015	8.2%	0.69 [0.31, 1.54]	2015
Robinson_12wk (R) 2015	17.4%	1.52 [0.90, 2.55]	2015
Takekita_12wk (Per) 2015	5.3%	2.44 [0.88, 6.82]	2015
Pagsberg_12wk (Q) 2017	43.5%	1.39 [1.08, 1.79]	2017
Wang_7wk (R+Q+O+Z) 2017	8.5%	2.12 [0.96, 4.68]	2017
Gomez-Revuelta_3yr (R+Q+O+Z) 2020	16.4%	1.24 [0.72, 2.12]	2020
**Subtotal (95% CI)**	**100.0%**	**1.42 [1.11, 1.81]**	
Total events			
Heterogeneity: Tau^2 = 0.02; Chi^2 = 7.25, df = 6 (P = 0.30); I^2 = 17%			
Test for overall effect: Z = 2.79 (P = 0.005)			
**1.8.2 Short-term studies**			
Maat (R) 2014	0.4%	9.92 [0.55, 178.45]	2014
Takekita_12wk (Per) 2015	3.5%	2.44 [0.88, 6.82]	2015
Robinson_12wk (R) 2015	13.8%	1.52 [0.90, 2.55]	2015
Pagsberg_12wk (Q) 2017	58.4%	1.39 [1.08, 1.79]	2017
Juncal-Ruiz_6wk (R+Q+O+Z) 2017	17.9%	1.39 [0.88, 2.20]	2017
Wang_7wk (R+Q+O+Z) 2017	5.9%	2.12 [0.96, 4.68]	2017
**Subtotal (95% CI)**	**100.0%**	**1.49 [1.23, 1.80]**	
Total events			
Heterogeneity: Tau^2 = 0.00; Chi^2 = 3.88, df = 5 (P = 0.57); I^2 = 0%			
Test for overall effect: Z = 4.04 (P < 0.0001)			
**1.8.3 Long-term studies**			
Savitz_26wk (Pal) 2015	36.5%	0.69 [0.31, 1.54]	2015
Gomez-Revuelta_3yr (R+Q+O+Z) 2020	63.5%	1.24 [0.72, 2.12]	2020
**Subtotal (95% CI)**	**100.0%**	**1.00 [0.57, 1.74]**	
Total events			
Heterogeneity: Tau^2 = 0.05; Chi^2 = 1.42, df = 1 (P = 0.23); I^2 = 30%			
Test for overall effect: Z = 0.01 (P = 0.99)			

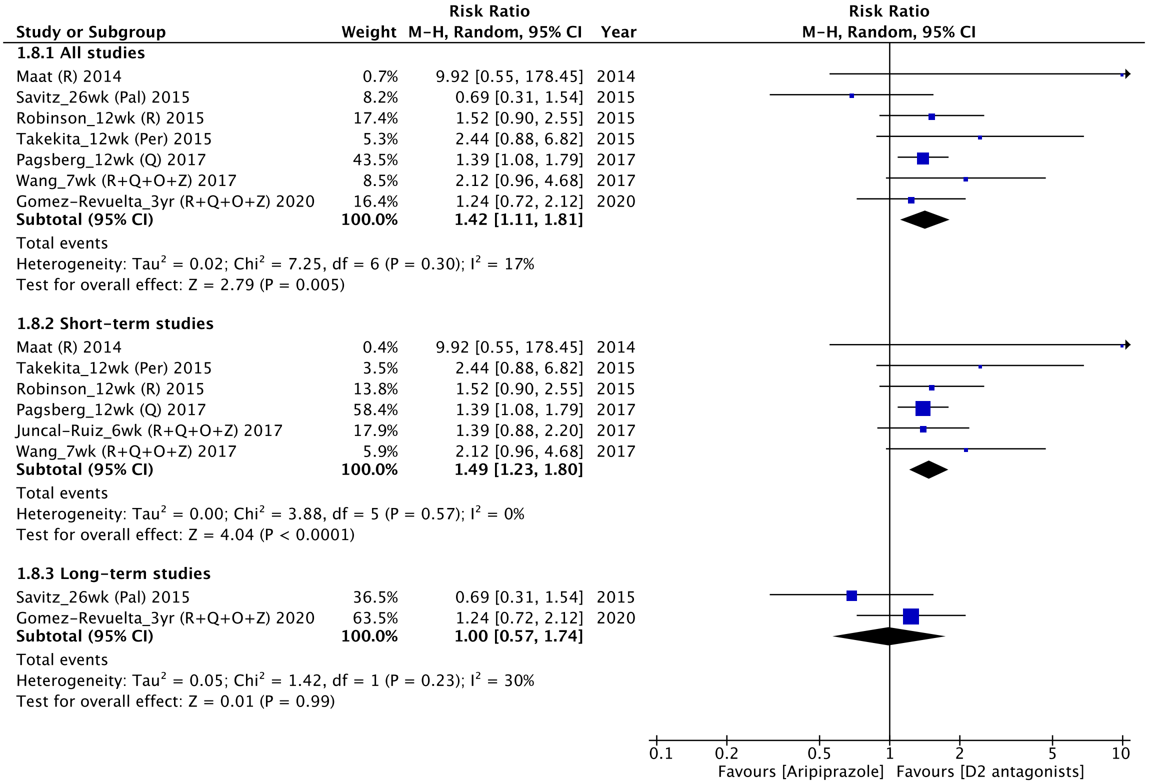

Supplementary Figure 3. Forest plots of comparisons of aripiprazole with D_2R antagonists for main findings. H: haloperidol; O: olanzapine; Pal: paliperidone; Per: perospirone; Q: quetiapine; R: risperidone; Z: ziprasidone. For plots i) and j), haloperidol trials were removed for a more meaningful comparison of aripiprazole with second-generation D_2 antagonists.

Supplementary Table 1. Meta-regression analysis of the association of covariates with overall symptom reduction and all-cause discontinuation

	N	Estimate	SE	*t*	95% CI	*p*
Overall symptom reduction						
Sample size	14	0.00	0.00	-0.42	-0.01 to 0.00	0.679
Percent open label	13	-0.19	0.29	-0.67	-0.83 to 0.45	0.519
Study year	14	-0.01	0.05	-0.19	-0.13 to 0.10	0.851
Age	13	0.02	0.02	1.21	-0.02 to 0.06	0.250
Percent male	13	0.00	0.01	-0.18	-0.02 to 0.02	0.859
Trial duration	14	-0.01	0.00	-2.69	-0.01 to -0.00	**0.020**
Percent first episode	14	0.22	0.26	0.83	-0.35 to 0.79	0.421
Aripiprazole dose	13	-0.01	0.02	-0.53	-0.06 to 0.04	0.609
Baseline PANSS total	14	-0.02	0.01	-2.30	-0.03 to -0.00	**0.041**
Percent risperidone/olanzapine	14	-0.16	0.30	-0.55	-0.81 to 0.49	0.594
Blinding[a]	13	-0.07	0.15	-0.46	-0.41 to 0.27	0.653
All-cause discontinuation						
Sample size	11	-0.00	0.00	-0.78	-0.01 to 0.00	0.435
Percent open label	11	-0.06	0.29	-0.20	-0.63 to 0.51	0.838
Study year	11	0.01	0.05	0.29	-0.08 to 0.10	0.775
Age	11	-0.01	0.02	-0.34	-0.06 to 0.04	0.736
Percent male	11	-0.00	0.01	-0.36	-0.02 to 0.02	0.717
Trial duration	11	-0.00	0.00	-0.62	-0.01 to 0.00	0.534
Percent first episode	11	0.13	0.30	0.42	-0.46 to 0.71	0.676
Aripiprazole dose	10	-0.02	0.01	-1.46	-0.05 to 0.01	0.146
Baseline PANSS total	11	-0.01	0.01	-1.44	-0.03 to 0.00	0.149
Percent risperidone/olanzapine	11	0.01	0.30	0.03	-0.58 to 0.60	0.974
Blinding[a]	11	-0.04	0.15	-0.26	-0.33 to 0.25	0.796

[a]Open-label, single-blind, and double-blind studies coded as 0, 1, and 2, respectively. D_2R: dopamine D_2 receptor; PANSS: Positive and Negative Syndrome Scale.

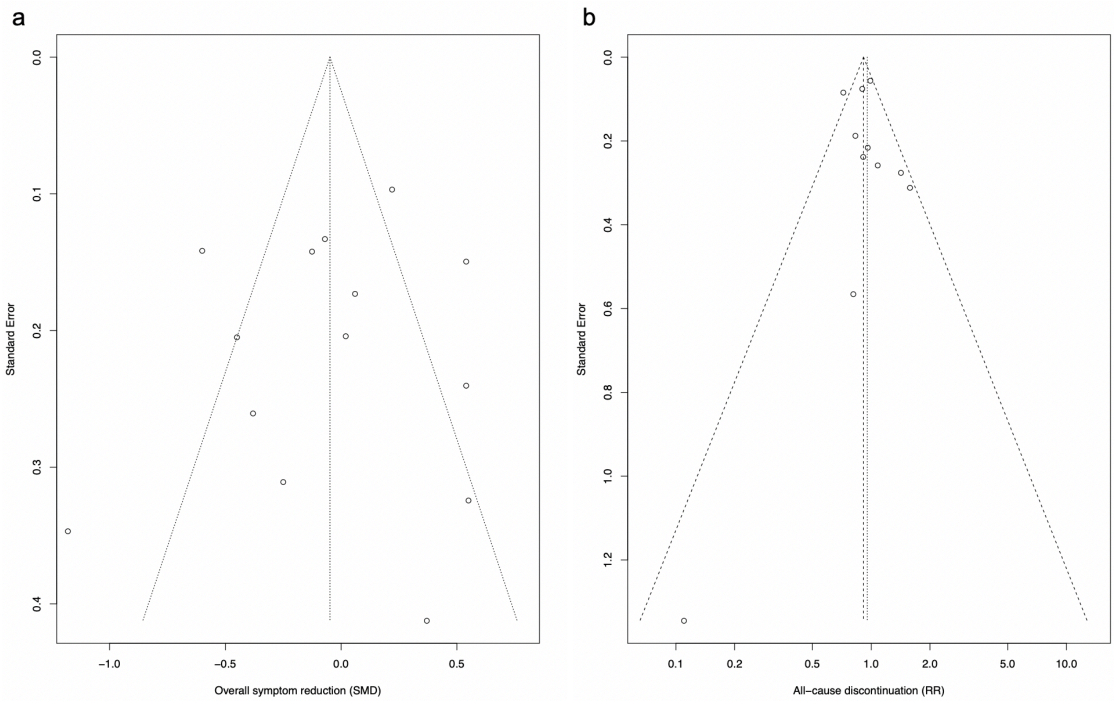

Supplementary Figure 4. Funnel plots of a) overall symptom reduction and b) all-cause discontinuation.
